# Structural assembly of the tailed bacteriophage ϕ29

**DOI:** 10.1038/s41467-019-10272-3

**Published:** 2019-05-30

**Authors:** Jingwei Xu, Dianhong Wang, Miao Gui, Ye Xiang

**Affiliations:** 1 Beijing Advanced Innovation Center for Structural Biology, Beijing Frontier Research Center for Biological Structure, Center for Infectious Disease Research, Department of Basic Medical Sciences, School of Medicine, Tsinghua University, 100084 Beijing, China; 2 Present Address: Institute of Molecular Biology and Biophysics, Eidgenössische Technische Hochschule Zürich, CH-8093 Zürich, Switzerland; 3 Present Address: Department of Biological Chemistry and Molecular Pharmacology, Harvard Medical School, Boston, MA 02115 USA

**Keywords:** Cryoelectron microscopy, Bacteriophages

## Abstract

The mature virion of the tailed bacteriophage ϕ29 is an ~33 MDa complex that contains more than 450 subunits of seven structural proteins assembling into a prolate head and a short non-contractile tail. Here, we report the near-atomic structures of the ϕ29 pre-genome packaging head (prohead), the mature virion and the genome-emptied virion. Structural comparisons suggest local rotation or oscillation of the head-tail connector upon DNA packaging and release. Termination of the DNA packaging occurs through pressure-dependent correlative positional and conformational changes in the connector. The funnel-shaped tail lower collar attaches the expanded narrow end of the connector and has a 180-Å long, 24-strand β barrel narrow stem tube that undergoes conformational changes upon genome release. The appendages form an interlocked assembly attaching the tail around the collar. The membrane active long loops at the distal end of the tail knob exit during the late stage of infection and form the cone-shaped tip of a largely hydrophobic helix barrel, prepared for membrane penetration.

## Abstract (web summary)

Mature particles of bacteriophage ϕ29 consist of a 33-MDa complex formed by over 450 subunits, assembled into a head and a short tail. Here, Xu et al. report the near-atomic structures of the ϕ29 prohead, the mature virion and the genome-emptied virion, providing insights into DNA packaging and release.

## Introduction

Tailed bacterial viruses (bacteriophages) are mega macromolecular complexes assembled from hundreds of subunits. The capsid heads of tailed bacteriophages are stable isometric or prolate protein shells that protect viral genomes and are resistant to harsh environmental conditions. Bacteriophage tails are the machinery responsible for specific host-cell recognition and infection. The tail attaches to the head at a special vertex of the capsid where a pentameric capsomer is replaced by a protein assembly known as the connector or portal^1^. The head and tail have different symmetries and have been suggested to assemble in a fixed relationship to form the complex viral machine^2–4^.

**Fig. 1:**
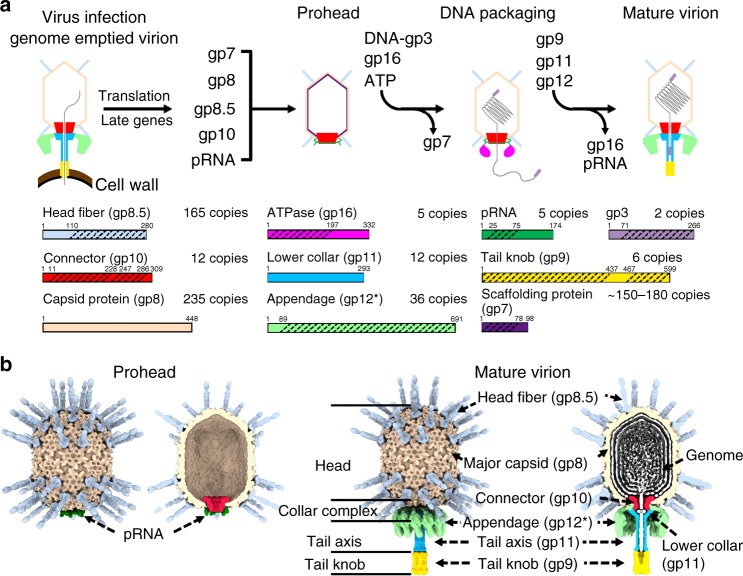
Schematic diagrams showing the life cycle, key particles, and structural components of the bacteriophage ϕ29. **a** Top: A schematic diagram showing the life cycle of ϕ29; bottom: diagrams showing the polypeptide chains of the structure components. The oblique dashed lines indicate the portions for which the atomic structures are available. **b** Surface shadowed diagrams show key particles of ϕ29. A cross-section is shown at the right side of each particle. The head fibers are colored cyan. The major capsids are colored brown. The connectors are colored red. The low collars are colored light blue. The appendages (tail spikes) are colored green. The tail knobs are colored yellow

The bacteriophage ϕ29, a representative of bacteriophages with a short non-contractile tail, has an ~19 kb dsDNA genome encoding ~20 proteins. Both 5ʹ ends of the genome are covalently connected to the terminal protein gene product 3 (gp3)^5^. ϕ29 infects the Gram-positive *Bacillus subtilis* cells by injecting the genome–gp3 complex into the host-cell cytoplasm using a short non-contractile tail, leaving the genome emptied virion on the cell surface (Fig. 1a). The translation of late phage genes, including the major capsid protein gp8, the head fiber protein gp8.5, the scaffolding protein gp7, and the connector protein gp10, initiate the assembly of an empty pro-capsid shell (prohead) (Fig. 1)^6^. A dsDNA packaging machinery is assembled on the prohead by attaching a viral encoded short prohead RNA (pRNA) and the ATPase gp16 around the connector^7–9^. The genome dsDNA–gp3 complex can be packaged by the motor through a channel of the connector into the empty prohead^7,10^. Low-resolution structural data showed only 5 pRNAs on each prohead^8,11^ (Fig. 1b). However, the copy number of the pRNAs was a topic of debate^12,13^. Early crystal structural studies of the scaffolding protein gp7 showed that two gp7 molecules form an arrow-like homodimer^14^. The location of the scaffolding proteins in the head has not been clearly defined. The pRNA and the gp16 ATPase disassociate from the head upon the triggering of an as yet unclear head-full signal. The tail proteins gp11, gp9, gp12, and gp13 then sequentially attach to form the mature virions (Fig. 1).

Previous low-resolution electron microscopy (EM) structural studies showed that the major capsid proteins of the mature ϕ29 head assemble to form a *T* = 3, *Q* = 5 prolate icosahedron^2,15–17^ (Fig. 1b). Low-resolution architecture of the major capsid protein gp8 suggested that it adopts a HK97 fold^18^ with an additional C-terminal immunoglobin like (Ig-like) domain^16^. However, atomic structures of the ϕ29 capsids are still missing. There are 55 head fibers protruding on the head (Fig. 1b). Each of the head fibers is a homotrimer of gp8.5^16,19^, which consists of a pseudo-hexagonal base and a protruding stem with a unique three-stranded helix-turn-helix supercoil structure^19^. Structural details of the pseudo-hexagonal base and its interaction with gp8 are under determination. Crystal structure of the connector showed that it is a dodecamer of gp10 with a central channel ranging from ~37 Å at the narrow end to 60 Å at the wide end^11^. The connector narrow end forms a bulge with the lower collar protein gp11 and has a different conformation with the tail attached^2,17^. Following the bulge is the tail axis or tail tube, which is also part of the lower collar protein gp11 (Fig. 1b). Gp11 was suggested to play an essential role in holding the terminus of the genome^20^. However, gp11 shares no sequence similarities to other tube forming proteins, thus little information is known about the molecular architecture of the tail tube and its interaction with the connector. Each appendage (tail spike) is a homotrimer of gp12* and is responsible for host-cell recognition^21^. Twelve appendages hang around the exterior surface of the bulge through 12 arms^2^ (Fig. 1b). Crystal structure of the C-terminal receptor recognition domain of gp12* has been reported previously^21^, whereas atomic structures of the N-terminal arms have not been determined and attachment of the appendages to the tail is not clearly understood. The distal end of the tail axis is attached with the tail knob, which is mostly assembled by the tail protein gp9. The tail lysozyme-like protein gp13 has been suggested to be part of the tail knob^22,23^. Gp9 contains a hydrophobic pore-forming loop (L loop) for breaching the host-cell membrane during infection^24^ (Fig. 1). Atomic details of the post genome injection gp9 assembly are still missing.

Here, we report the cryo-electron microscopy (cryo-EM) structural studies of the ϕ29 prohead, the mature and the genome-emptied virions at near-atomic resolutions up to 3.2 Å. Full atomic models of the three complex virions were built. The complete mature and the genome-emptied virion models each consist of 235 copies of the major capsid protein gp8, 165 copies of the minor capsid protein gp8.5, 12 copies each of the connector protein gp10 and the tail protein gp11, 36 copies of the tail appendage protein gp12* and 6 copies of the tail knob protein gp9. The prohead model includes 5 copies of the pRNA, 110 copies of the scaffolding protein gp7, and the same copy numbers of the capsid and connector proteins as these of the mature or the genome-emptied particles. Of note, through structural comparisons we found that the connector was pushed out of the capsid for ~13 Å during head maturation, suggesting a pressure-dependent mechanism of DNA packaging termination. In addition, the tail tube structure determined shows a unique β-barrel structure. The tail appendages form around the tail bulge an interlocked assembly that may facilitate a synergetic sense of host-cell receptors.

## Results

### The capsid protein structure

We calculated the cryo-electron microscopy (cryoEM) reconstructions of the ϕ29 prohead, the mature head and the genome emptied head to a resolution of 3.6, 3.2, and 3.2 Å, respectively (Supplementary Figs. 1 and 2, Methods). The final maps were sufficient for ab initio model building of the capsid shell. Atomic models were built for the three head capsids (Supplementary Table 1).

**Fig. 2:**
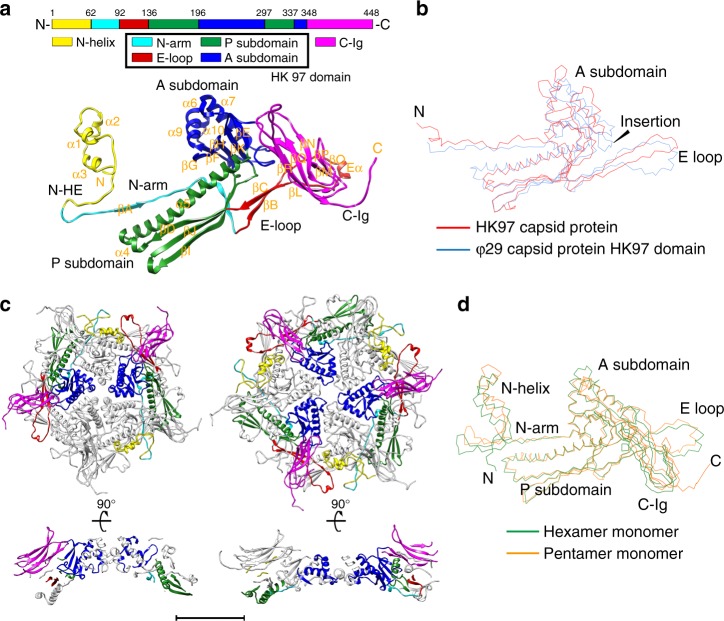
Structure of the mature capsid. **a** Structure of the mature virion major capsid protein gp8. Top: a schematic diagram showing the polypeptide chain of a gp8 monomer; bottom: a ribbon diagram of a gp8 monomer structure in a pentameric capsomer. The structural domains of gp8 are shown in yellow (N-helix), cyan (HK97-N arm), red (HK97-E loop), green (HK97-P), blue (HK97-A), and magenta (C-Ig). **b** Structural superposition of the ϕ29 HK97 domain (blue) and the HK97 capsid protein (red). **c** Top and side view ribbon diagrams of a pentameric capsomer and a hexameric capsomer. The structural domains of two gp8 monomers in the pentameric capsomer and three gp8 monomers in the hexameric capsomer are colored in the same manner as in **a**. The scale bar represents 5 nm. **d** Structural superposition of a pentameric capsomer monomer (orange) and a hexameric capsomer monomer (green)

Our results showed that the 448-amino-acid-long polypeptide chain of the major capsid protein gp8 folds into three structural domains: the small N-terminal helix domain (the gp8-N-helix domain, residues 1–61), the central HK97 fold domain (the gp8-HK97 domain, residues 62–347) and the C-terminal immunoglobulin-like domain (the gp8-C-Ig domain, residues 348–448) (Fig. 2a and Supplementary Fig. 3a). The structure of the central HK97 domain closely resembles that of the HK97 capsid protein, which has two subdomains (the axial subdomain A and the peripheral subdomain P) with extensions (N-arm and E-loop) (Fig. 2a). Superposition of the gp8-HK97 and the HK97 capsid structures (PDB entry 1OHG, Chain A, 10.2210/pdb1OHG/pdb)^18^ showed a r.m.s.d. of 2.7 Å between 202 aligned equivalent Cα atoms (Fig. 2b). Nevertheless, the sequence identities of the aligned residues are only 22.7%. The gp8-N-helix domain contains ~60 amino acids that mainly fold into three short helices (Nα1–3) and a 27 Å-long extension (N-helix extension, N-HE). The extension forms a hairpin structure with the distal end of the HK97 domain N-arm. The gp8-C-Ig is an all β-strand immunoglobulin-like domain, which is connected to gp8-HK97 through a linker of four flexible residues (Ser343Gly344Asp345Val346). Dali search of homologous structures showed that the structure of gp8-C-Ig is closely similar to that of a bacterial protein with a predicted function of cellulose binding (*z-*Score: 8.9, PDB entry 1F00, 10.2210/pdb1F00/pdb)^25^.

### The capsid assembly

**Fig. 3:**
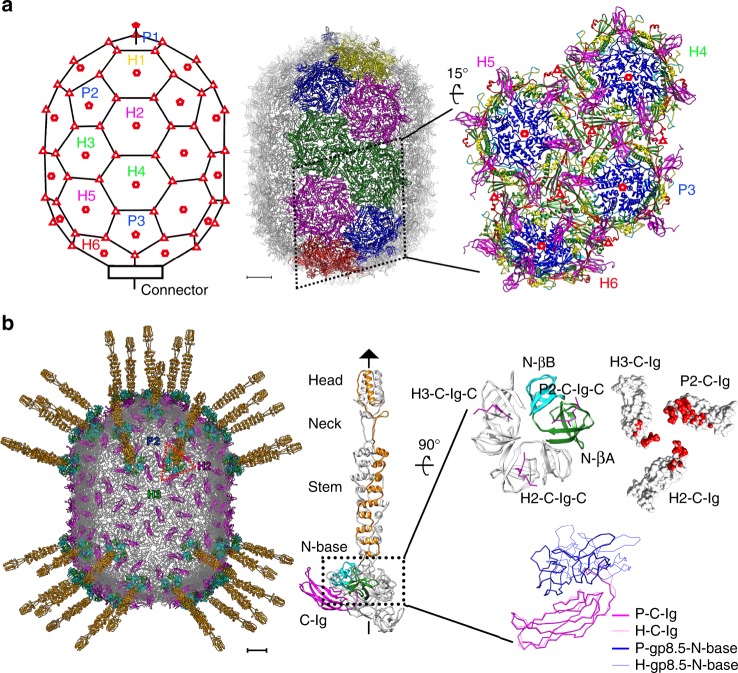
Capsid assembly of the mature head. **a** Left: A schematic diagram showing the capsomer organization of the head. Capsomeres in an asymmetric unit are labeled. Capsomeres with a similar environment are labeled in the same color. Quasi sixfold, fivefold, and threefold positions are indicated with hollow hexagons, pentagons, and triangles, respectively; middle: ribbon diagram structure of the mature virion head. Capsomeres in an asymmetric unit of the head are colored. Capsomeres in a similar environment are colored the same; right: A zoom-in ribbon diagram of the capsomeres in half of the asymmetric unit showing the interactions between the capsomeres. The structure domains of each monomer are colored the same as in Fig. 2a. The scale bar represents 5 nm. **b** Left: Ribbon diagrams showing the head fiber structures at the quasi threefold positions. The fibrous part of each head fiber is colored orange. The βA and βB subdomains of each gp8.5-N in the fiber base are colored green and cyan, respectively. The C-Igs of the major capsid protein are colored magenta. The rest of the major capsid protein is colored gray; middle: ribbon diagrams showing the structure of a head fiber trimer in complex with 3 C-Igs. One gp8.5 monomer of the head fiber and one of the C-Igs are colored the same as in the left diagram; right-top: ribbon and surface shadowed diagrams showing the interactions between the gp8.5-N fiber base and the C-Igs. The contact area is colored red; right-bottom: structural comparisons of the gp8.5-N (blue, thick lines) on a C-Ig (magenta, thick lines) from a pentameric capsomer and the gp8.5-N (blue, thin lines) on a C-Ig (magenta, thin lines) from a hexameric capsomer. The scale bar represents 5 nm

The total 235 gp8 subunits assemble into a ~520 Å long and 400 Å wide, *T* = 3, *Q* = 5 prolate icosahedron of 30 flat hexameric capsomeres and 11 concave pentameric capsomeres (Figs. 1b, 2c, and 3a). Structural superposition showed that the gp8 monomers from the two different types of capsomeres differ in the N-helix domain and the E loop of the HK97 domain (Fig. 2d). Structural comparisons using the HK97 domains showed significantly greater variation in the hexameric capsomeres than that in the pentameric capsomeres (an average r.m.s.d. of 2.2 Å vs. 0.6 Å between the aligned Cα atoms of the corresponding HK97 domains of the mature head capsomeres) (Supplementary Table 2). The hexameric capsomeres of the icosahedral end caps (H1, H2, H5) show features of a trimer, whereas the ten equatorial hexameric capsomeres (H3, H4) have the largest distortion and show features of a dimer (Fig. 3a and Supplementary Table 2).

Gp8 subunits within the hexameric or pentameric capsomeres are associated mainly through the interactions between the A and P subdomains of the HK97 domains (Fig. 2c and Supplementary Table 3). In addition, extensive domain swaps between the HK97 domains enhance the stability of the capsomer. The E loop spans the exterior surface of the HK97-P subdomain from a neighboring monomer within the capsomer. The neck of the E loop forms a short anti-parallel β-sheet with the N-arm from the same neighboring monomer (Fig. 2c). Most of the N-arm in the monomer is associated with the interior surface of a different neighboring monomer. The N-terminus of the HK97 N-arm extends outwards to the exterior surface of the capsid, wraps 180° around the neck region of a neighboring E-loop to form the hairpin structure with the N-helix extension and redirects the entire N-helix domain to the exterior surface of the same neighboring HK97-P subdomain. The three short helices of the N-helix domain form a helix bundle together with the long helix of the neighboring HK97-P subdomain, thus firmly fixing the hairpin structure in place (Fig. 2a, c).

Interactions between the capsomeres are mainly mediated by the HK97-P subdomains. Additional interactions with neighboring capsomeres are made through the tips of the E loops, part of the N hairpins and the C-Igs (Fig. 3a and Supplementary Table 3). The root of the C-Ig protrudes near the center of the capsomeres. Except for the gp8 monomers proximal to the connector protein, the polypeptide linker connecting the C-Ig turns 180º around near the center of each capsomer, which points the C-Ig outwards to the outer edge of the capsomer to form axial ridges on the exterior capsid surface (Fig. 3a). The outer surface of the capsid is largely negatively charged (Supplementary Fig. 3b). However, the inner surface of the capsid is largely neutral and is decorated with well-dispersed negatively and positively charged residues (Supplementary Fig. 3b).

### The head fiber structure

With the cryoEM map of the mature head calculated at a resolution of 3.2 Å (Supplementary Table 1), we determined the structure of the pseudo-hexagonal base of the head fiber (residues 1–110 of gp8.5). The results showed that the N-terminal 110 residues of gp8.5 form two small β-barrel domains (gp8.5-N-βA and N-βB) connected by a short linker (residues 58–61) (Fig. 3b). The two small β-barrel domains are similar to each other. Six β-barrel domains from three gp8.5 monomers form the pseudo-hexagonal base of each head fiber, which interact with one C-Ig from a pentameric capsomer with a contact surface area of ~620 Å^2^ and two C-Igs each from a different hexameric capsomer with an average contact surface area of ~400 Å^2^ (Fig. 3b). The monomers in the trimer are associated with each other through hydrophobic interactions and have large buried surfaces in between them. We also determined the crystal structure of the N-terminal pseudo-hexagonal base (gp8.5 N-base, residues 1–116) to a resolution of 1.8 Å by molecular replacement using the EM map as a search model (Table 1). The crystal structure shows a similar homotrimer with a β-strand missing from each gp8.5-N-βA domain of the trimer. Further analysis showed that the missing β-strands are complemented by the C termini of the three C-Igs in the assembled gp8.5 N-base (Fig. 3b).

**Table 1:** X-ray crystallographic data collection and refinement statistics for gp8.5-N

Wavelength (Å)	0.9798
Resolution range (Å)^a^	41.79–1.80 (1.864–1.80)
Space group	*P2*_*1*_
Unit cell: *a*, *b*, *c*, *α*, *β*, *γ*	55.212, 65.07, 118.874, 90, 99.091, 90
Unique reflections^a^	76833 (7633)
Multiplicity^a^	3.4 (3.2)
Completeness (%)^a^	99.31 (99.18)
Mean I/sigma^a^	13.5 (1.5)
R-merge^a^	0.087 (0.376)
Wilson B-factor (Å^2^)	29.60
R-work^a^	0.1825 (0.3191)
R-free^a^	0.2245 (0.3526)
Number of non-hydrogen atoms	6017
Macromolecules	5394
Ligands	40
Solvent	583
RMSD(bonds)	0.006
RMSD(angles)	0.87
Ramachandran favored (%)	96.99
Ramachandran outliers (%)	0.00
Average B-factor (Å^2^)	41.77
Macromolecules	40.52
Solvent	48.27

^a^Statistics for the highest-resolution shell are shown in parentheses

### Structure of the scaffolding and comparisons of the heads

**Fig. 4:**
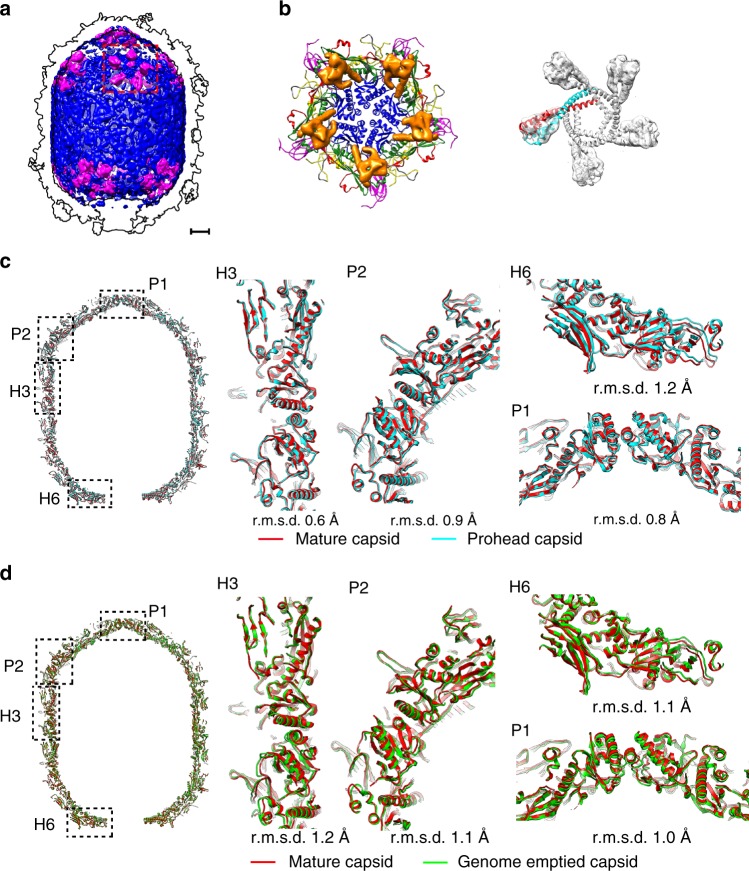
Structure of the scaffolding protein and structural comparisons of the capsids. **a** Surface shadowed diagrams showing the internal scaffolding proteins of the prohead (magenta). The weak density shell in the prohead capsid is colored blue. The outline of the capsid is indicated with black lines. The scale bar represents 5 nm. **b** Ribbon and shadowed-surface diagrams showing the attachment of five scaffolding dimers (surface shadowed and colored orange) to a pentameric capsomer (in ribbon); right: fitting of the scaffolding dimers into the EM density map. **c** Structural comparisons of the prohead (blue) and the mature head (red) capsids. Left: overall comparison; middle and right: zoom-ins showing the subtle differences at different parts of the head. The scale bar represents 5 nm. **d** Structural comparisons of the mature head (red) and the genome emptied head (green) capsids. Left: overall comparison; middle and right: zoom-ins showing the subtle differences in different parts of the head. The scale bar represents 5 nm

After having built the atomic model of the prohead capsid, we observed un-interpreted weak densities that do not belong to the capsid underneath the pentameric capsomeres. Local averaging of the densities showed features consistent with the arrow shaped scaffolding dimers (Fig. 4a, b). One long helix of the scaffolding dimer is associated with the long helix of the HK97-P subdomain (α5) (Fig. 4b), which is in agreement with previous biochemical analysis^26^. The arrowhead of the scaffolding dimer attaches a featureless weak density shell inside the capsid (Fig. 4a, blue). These densities were not observed in the mature capsid (Supplementary Fig. 3c).

The capsid proteins are densely packed in all the heads, leaving tiny channels that could allow the passage of ions (Supplementary Fig. 3d). Structural comparisons of the heads showed overall small differences between the heads (an average r.m.s.d of 0.7 Å, Supplementary Table 4). The differences between the equatorial parts of the prohead and the mature head are smaller than those between the icosahedral end caps (an average r.m.s.d of 0.5 Å vs. 0.8 Å, Supplementary Table 4). Further analysis showed that the icosahedral end caps of the mature head expanded slightly upon the loading of the genome (Fig. 4c). However, structural comparisons of individual hexameric and pentameric capsomeres within the icosahedral end caps showed a similar average r.m.s.d. of 0.4 Å (Supplementary Table 4), indicating that the expansion in the icosahedral end caps mainly occurs through the rigid-body movements of the capsomeres. In comparison with most hexameric capsomeres except for the one near the connector (H6), the pentameric capsomeres exhibit a greater shift outwards (Figs. 3a, 4c and Supplementary Table 4). Structural comparisons of the mature and the genome-emptied heads showed that the equatorial part of the genome-emptied head expanded slightly after the ejection of the genome (Fig. 4d and Supplementary Table 4). However, the icosahedral end caps shrink upon the release of the inner pressure, showing the elasticity of the capsid.

### The connector structure

**Fig. 5:**
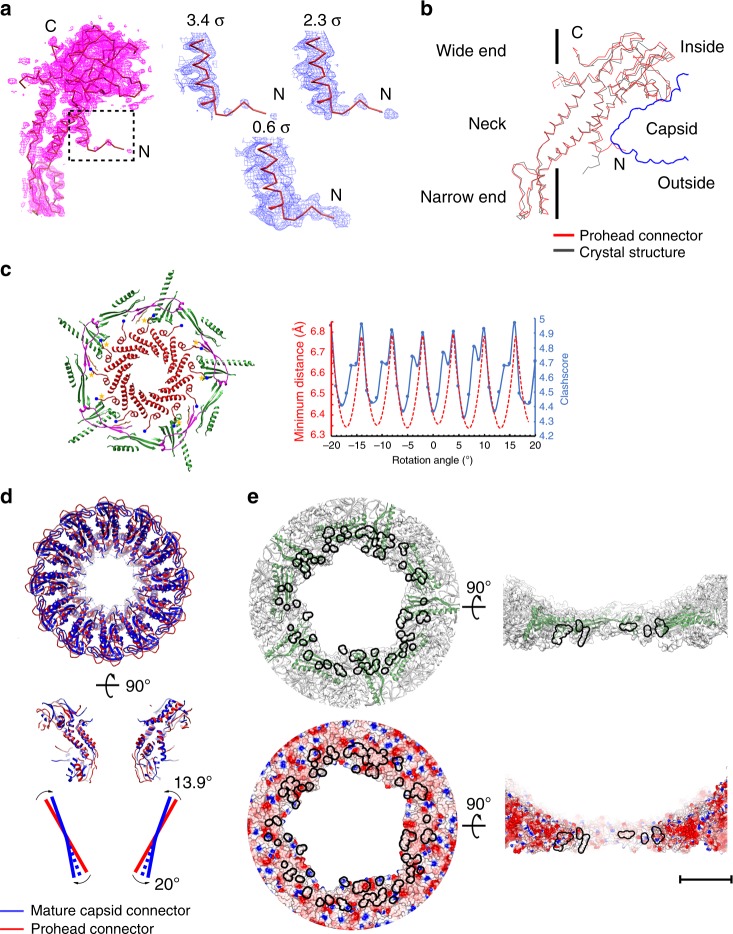
Structure of the connector. **a** Left: a wire-frame structural model of a gp10 monomer shown in the prohead connector density. The density map of the prohead connector is drawn at a contouring level of 3.4 σ (magenta); right: zoom-ins showing the extended distal end of gp10 N-terminus at three different contouring levels. **b** Structure superposition of a prohead connector monomer (red) and a crystal structure connector monomer (black). The vertical black line indicates the position of the symmetry axis. **c** Left: ribbon diagrams showing the interactions between the prohead capsid and the gp10 N termini of a randomly placed prohead connector in a fivefold reconstructed prohead connector envelope. The connector is colored red. The N termini of the gp10 monomers are marked with blue balls. Yellow stars indicate gp10 monomers that have steric clashes with the capsid. The HK97-P subdomains are colored green. The E loops are colored magenta; right: plots of the clashscores (blue solid line) and the minimum distances (red dash line) against the rotational positions of the connector. The connector positions were generated by rotating the connector around its 12-fold symmetry axis with a step size of 1 degree. The clashscore calculated using the program phenix.molprobity is defined as 1000 × (number of bad overlaps)/(number of atoms). The minimum distance at each position was calculated using 12 Tyr122 Cα atoms of the connector and 5 Trp156 Cα atoms of the capsid. **d** Structure superposition of the prohead connector (red) and the mature head connector (blue) showing conformational changes of the connector upon head maturation. Top: top view; middle: side view; bottom: a schematic diagram showing possible level motion of the gp10 monomers. **e** Ribbon and shadowed-surface diagrams showing the contact sites of the connector to the mature capsid. The contacts are highlighted with black lines. Top: ribbon and shadowed-surface; bottom: electrostatic surface. Negative and positive electrostatic potentials are colored red and blue, respectively. The scale bar represents 5 nm

By using focused refinements and reconstructions on the connector, we determined the in situ atomic structures of the connectors (Fig. 5a–c and Supplementary Fig. 2b, Methods), which allowed us to perform a detailed analysis of the interactions with the capsid and conformational changes of the connector during virus maturation and infection. The gp10 connector dodecamer is surrounded by ten HK97-P subdomains from five hexameric capsomeres. Five of the ten HK97-P subdomains are oriented radially to the connector, whereas the other five of the ten HK97-P subdomains are oriented tangentially to the connector (Fig. 5c). The neck of the connector has close contacts with the gp8-N arms and the gp8-α4s of the five radially oriented HK97-P subdomains, which are similarly disordered in all the head reconstructions assuming fivefold symmetry (Supplementary Fig. 4a). Empty spaces are between the neck and the five tangentially oriented HK97-P subdomains (Supplementary Fig. 4a).

The in situ prohead connector structure closely resembles the crystal structure of the gp10 dodecamer with differences in the N-terminal helix (gp10-α1) and loops of the wide and narrow ends (Fig. 5b). Gp10-α1 is a long helix of six helix turns in the crystal structure^11^. The reconstructed density of the prohead connector assuming 12-fold symmetry shows that the distal end one and half helix turns of gp10-α1 disassemble and radially protrude outward for ~15 Å (Fig. 5a, b). However, placement of the prohead connector structure in the capsid showed that some of the protruding gp10 N termini have steric clashes with the N-arms, E-loops and the radially oriented HK97-P subdomains (Fig. 5c). Steric clashes are avoided only for these located in the empty spaces between the neck and the tangentially oriented HK97-P subdomains. Thus, the protruding N termini of gp10s should prevent free rotation of the connector. However, local rotation or oscillation of the connector within the capsid is still possible (Fig. 5c).

**Fig. 6:**
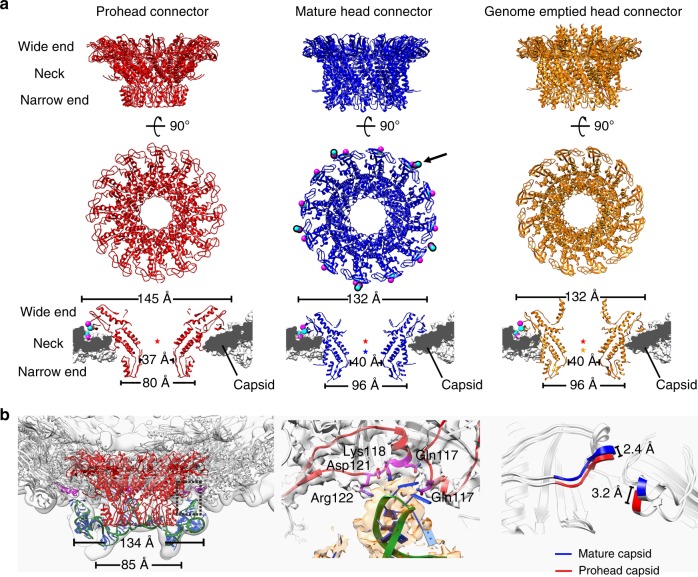
Structural comparisons of the connectors. **a** Top and middle: ribbon diagrams showing the top and side views of the prohead (red), the mature head (blue) and the genome emptied head connectors (orange); bottom: thin sections of different connector in corresponding capsid showing the conformational and positional differences between the connectors. Residues Arg94 and Glu103 of the mature head connector and residues Lys297 and Asp298 of the HK97-P subdomain are represented using balls (blue for Arg and Lys, magenta for Asp and Glu). Center of gravity for each connector is indicated with a star to allow comparison of the positions of the connectors relative to the capsid. **b** Left: ribbon diagrams showing the pRNAs on the prohead. The backbone of the pRNA is shown in green with the bases colored blue. The prohead connector is colored red. Residues in contact with the pRNA are shown in magenta; middle: interactions between the pRNAs and the capsid E loops. Residues in contact with the pRNA are colored magenta. The E loops are colored red. The pRNA density is shown in transparent orange; right: conformational changes of the E loops upon head maturation

The neck, the wide and narrow ends of the prohead connector have an outer diameter of 94, 145, and 80 Å, respectively. The connectors in the mature and the genome-emptied heads (post maturation connectors) are similar (a r.m.s.d. of 0.9 Å between 246 Cα atoms of aligned residues, residues 18–226 and 248–284). However, they differ dramatically from that of the prohead connector at the narrow and wide ends (mature virion connector vs. prohead connector, r.m.s.d.: 7.4 Å for the narrow end, 1.9 Å for the neck and 5.0 Å for the wide end) (Fig. 5d). Further analysis showed that both the mature and the genome emptied connector display an expanded narrow end, the outer diameter of which increased by 16 Å to 96 Å, whereas the wide end of the mature or the genome-emptied connector contracts for 13 Å to a reduced outer diameter of 132 Å compared with the prohead connector (Figs. 5d, 6a). In addition, the size of the inner channel is increased by 3 Å in diameter at the neck region for the post maturation connectors (Fig. 6a). Structural comparisons of individual gp10 monomers suggested that the conformation change of the connector could be partially attributed to a level motion with the neck region as a pivot point (Fig. 5d). The outer surface of the prohead connector narrow end is smooth and featureless. The expansion of the narrow end exposes buried hydrophobic residues and generates hydrophobic pockets and gaps (Supplementary Fig. 4b), providing attachment sites for the lower collar of the tail. The gp10-α1s of the mature and genome-emptied connectors consist of a long helix of six helix turns similar to that of the crystal structure. The C termini of connector gp10s are disordered in the crystal structure, as well as the prohead and the mature head. However, they are ordered in the genome-emptied head and form a helix barrel structure at the top of the inner channel (Fig. 6a).

The relative positions of the connectors in the capsids were estimated using the gravity centers of the connector necks after superimposing the capsids (Supplementary Table 4). The results show that the connector is ~13 Å outward in the mature head compared with the prohead (Fig. 6a and Supplementary Table 4). Similarly, the connector of the genome-emptied head is positioned ~11 Å outward compared with that of the prohead (Fig. 6a and Supplementary Table 4). As observed in the capsid comparisons, the HK97-P subdomains around the connector have only a vertical expansion of ~2.3 Å in the mature virion (Fig. 6b). Thus, the shifts of the post maturation connectors are significant relative positional changes of the connectors to the capsid rather than accompanying shifts with the capsid expansion. The bottom edge of the prohead connector wide end loosely stacks on the interior surface of the HK97-P subdomains. Vertical movement of the prohead connector to a similar position as in the mature head would generate steric clashes between the wide end and the capsid, suggesting that the connector must undergo conformational changes to be adapted to a similar position as in the mature head. The wide end of the mature head connector has close contacts with the HK97-P subdomains (Figs. 5e, 6a), in which Glu103 and Lys94 of the connector wide end and Lys297 and Asp298 of the HK97-P subdomain form two salt bridges anchoring the connector to the capsid at a fixed position (Fig. 6a and Supplementary Fig. 4c). Although the structures of the mature and the genome-emptied connectors are highly similar, release of the inner pressure prompts vertical positional changes in the genome emptied connector that disrupt the salt bridges and free the connector for rotation or oscillation (Supplementary Fig. 4c). Consistent with the observed structures, analysis of the relative orientation between the connector and the capsid indicated that the connector does not have a fixed relative position to the pro- or the genome-emptied capsid, whereas the mature virion exhibits a fixed relationship between the connector and the capsid (Supplementary Fig. 2e).

### The pRNA structure

We determined the structure of the pRNA ring at a resolution of 4.6 Å through focused classifications and refinements (Supplementary Figs. 1 and 5a, Methods). Five pRNA molecules and structural features of the RNA duplexes could be clearly identified in the ring of pRNA density (Supplementary Fig. 5a). An atomic model consisting of the 5ʹ 69 nts (26–94) of the pRNAs was built based on the reconstruction. Densities of the 5ʹ 25 nts (1–25) and 3ʹ 80 nts (95–174) of the pRNAs were not visible in the map, probably due to the flexibility of the corresponding nts. Our results showed that the five pRNAs assemble to form a pentameric ring structure around the narrow end of the connector (Fig. 6b and Supplementary Fig.  5a). The inner diameter of the pentameric pRNA ring is ~85 Å, which is slightly larger than the narrow end of the prohead connector (Fig. 6b). However, the pRNA assembly does not have any direct close contact with the connector. Instead, the upper protrusions of the pRNA ring show extensive interactions with the E loops of the capsid through three nucleotides (U_54_G_55_A_56_). At each site of attachment to the capsid, residues Gln117, Lys118, Asp121, and Arg122 of one E loop and residue Gln117 of a neighboring E loop have direct contacts with the pRNAs (Fig. 6b). Depending on the relative position of the connector, the N-terminal helix α1 of one connector monomer could be in close proximity to the upper inner surface of the pRNA ring. As have been shown in the head comparisons, the E loop in contact with the pRNA has an outward shift of ~3 Å in the mature head (Fig. 6b). The shift of the E loops enlarges the distance between two neighboring E loops, thus disrupting the interactions with the pRNAs. In addition, the pRNA ring (inner diameter: 85 Å) is unable to adapt the expanded narrow end of the connector (outer diameter: 96 Å).

### The genome organization

Structural comparisons of the mature and the genome DNA emptied virions yielded clear boundaries for the densities of the encapsulated genome, which show distinct features even though being fivefold averaged. Overall the portion of the genome close to the capsid is packed in layers (Supplementary Fig. 5b). The distance between the layers is ~20 Å, which is consistent with previous observations made for ϕ29 and other viruses^4,27,28^. The outmost layer of the genome has recognizable features. The genome in association with the lower icosahedral cap forms a concentric spool around the fivefold symmetry axis (Supplementary Fig. 5b). Two clear distinct ring structures are wrapped on the top of the connector wide end, which is consistent with previous observations (Supplementary Fig. 5b)^2^. However, the genome in association with the equatorial capsomeres is organized in oblique lines rather than concentric rings (Supplementary Fig. 5b). The positions of the genome density in the oblique lines agree well with the inner surface electrostatic potential features of the capsid (Supplementary Fig. 3b). The distal end of the genome is located near the junction of the tail tube and knob. Focused refinements on the distal end of the genome showed features similar to those of the gp3 crystal structure^29^ (Supplementary Fig. 5c). The cavity of the collar complex is occupied by a toroid density, located ~20 bp from the terminal protein, which has been interpreted as an extremely deformed dsDNA fragment that helps to retain the DNA in the head^17^ (Supplementary Fig. 5b–d). However, our reconstruction showed that the toroid density does not have any direct close contact with the collar (Supplementary Fig. 5d). Furthermore, focused refinement on the toroid density shows features of a possible protein–DNA complex rather than a coiled dsDNA fragment (Supplementary Fig. 5c, d). Mass spectral analysis of the mature virion showed that the mature virion contains the phage encoded single stranded DNA-binding protein gp5 and histone-like protein gp6, both of which were shown to bind the right end of the genome to initial genome replication^30–32^ (Supplementary Figs. 5d and 6a and Supplementary Table 5). Mixing the purified gp5 and gp6 with the right end fragments of the ϕ29 genome DNA formed a similar toroid structure (Supplementary Fig. 6b, c).

### Structure of the lower collar

**Fig. 7:**
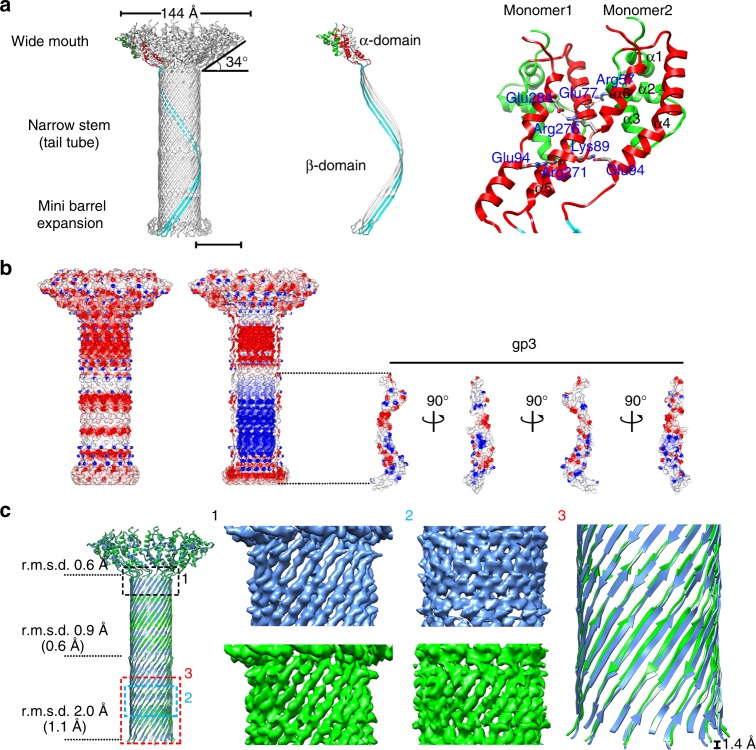
Structure of the lower collar. **a** Left: Ribbon diagrams showing the lower collar assembly; middle: ribbon diagrams showing two gp11 monomers of the lower collar assembly. One of the two monomers is colored according to its domains (gp11-α1–3: green, gp11-α4–6: red, β-domain: cyan), while the other gp11 monomer is colored gray; right: a zoom-in showing salt bridges in between two gp11 monomers at the wide mouth region of the funnel-shaped lower collar. The scale bar represents 5 nm. **b** Surface electrostatic potential of the lower collar and the terminal protein gp3. Negative and positive electrostatic potentials are colored red and blue, respectively. Left: outer surface electrostatic potential of the lower collar; middle and right: inner surface electrostatic potential of the lower collar and outer surface electrostatic potential of the terminal protein gp3. **c** Left: ribbon diagrams showing gp11 α-domain-based structural superimpositions of the mature virion lower collar (blue) and the genome emptied virion lower collar (green). R.m.s.d.s for different portions of the lower collar were calculated and shown at the left side of the superimposition. The r.m.s.d.s shown in the parentheses were calculated from the structural superimpositions by using the upper or the bottom portion of the β-domain; middle: representative densities of the upper and the bottom portions of the β-domains; right: a zoom-in showing the contraction of the lower collar upon genome release

**Fig. 8:**
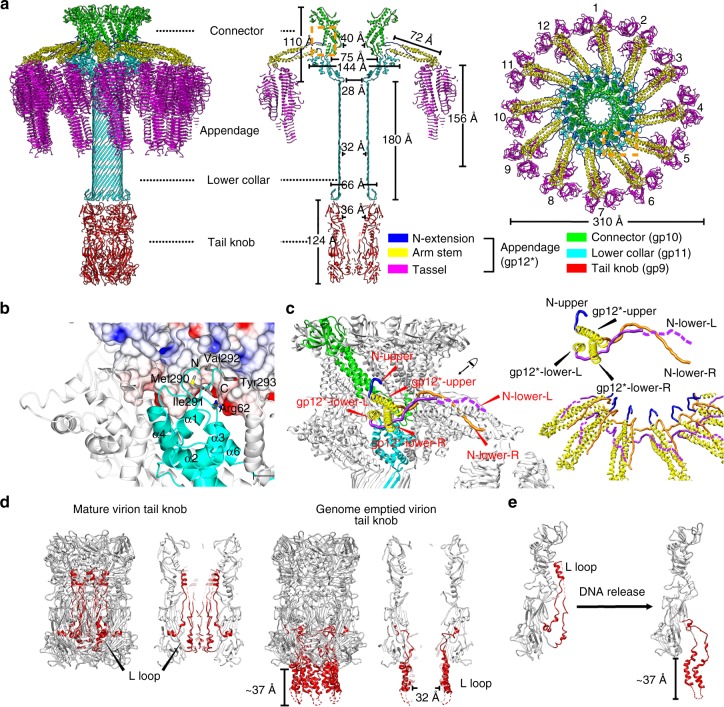
Structures and organizations of the collar complex and the tail knob. **a** Ribbon diagrams showing the overall organization and structure of the entire tail. Components of the tail are colored differently (connector: green, lower collar: cyan, N-extensions of tail appendages: blue, arm stems of tail appendages: yellow, tassels of tail appendages: magenta and tail knob: red). **b** Ribbon and shadowed-surface diagrams showing the interactions between the connector and the lower collar. The connector is shown in shadowed-surface colored by electrostatic potentials as in Fig. 5e. **c** Zoom-in showing the interlocked assembly of the tail appendages. Three N-terminus of each tail appendage are colored differently (N-upper: blue, N-lower-L: purple, N-lower-R: orange). The arm stems of the tail appendages are colored yellow. One connector monomer and one lower collar monomer are colored green and cyan, respectively. **d** Ribbon diagrams showing the tail knob structures. The hydrophobic L-loops are color red. The disordered regions of the L-loops (residues 440–455) are represented with dashed red lines. **e** Ribbon diagram showing the conformational change of L-loops upon genome release

Our results showed that the lower collar consists of 12 gp11 molecules, each of which consists of 293 amino acids assembling into a 12-fold-symmetric funnel-shaped structure (Fig. 7). The middle 156 residues (residues 103–258) of gp11 form two 180 Å long anti-parallel β-strands (gp11-βA and βD) (Fig. 7a and Supplementary Fig. 6d). Twenty-four anti-parallel β-strands are arranged in right-handed spirals that assemble to form the narrow stem of a 180 Å-long tube-like β-barrel with an outer diameter of 55 Å and an inner diameter of 32 Å (β-domain) (Figs. 7a, 8a). The two β-strands are connected at the distal end of the tube by a 16 amino acid loop (residues 172–187, gp11-βB and βC), which flips back to interact with the anti-parallel β-strands and forms a mini β-barrel (Fig. 7a and Supplementary Fig. 6d). The mini β-barrel generates an expansion around the distal end of the tube and mediates the interaction with the tail knob. The N-terminal 96 residues and the C-terminal 32 residues of gp11 fold into an elongated all α-structure (α-domain), among which residues 7–60 form a compact mini α-domain (gp11-mini α) consisting of three short helices (gp11-α1–3): residues 68–96 form a long helix (gp11-α4); and C-terminal residues 262–289 form two short helices (gp11-α5–6) aligned almost in parallel with the long helix gp11-α4 (Fig. 7a and Supplementary Fig. 6d). The parallel gp11-α4-α6s from twelve gp11s tilt ~56 degree away from the tail axis, assembling clockwise in a cone-shaped spiral wheel that constitutes the wide mouth of the funnel (Fig. 7a). The gp11-α1-α3s hang around the outer surface of the wide mouth. The widest outer and inner diameters of the open mouth are 144 Å and 75 Å, respectively. The interactions between the parallel gp11-αs are mainly salt bridges (Fig. 7a and Supplementary Table 3). The outer surface of the lower collar is largely negatively charged (Fig. 7b). The inner wall of the lower collar is rich in Arg and Lys and has distinct electrostatic features with alternative positively charged and negatively charged regions (Fig. 7b). The inner surface electrostatic potential of the tail tube is complementary to that of the terminal protein gp3 in the tube (Fig. 7b). Structural comparisons showed no significant differences between the wide mouths of the mature and the genome-emptied lower collars (r.m.s.d. 0.6 Å between Cα atoms of 134 aligned residues). However, the tube stem, especially of which the portion close to the tail knob, shrinks for ~1.4 Å upon the release of the genome (Fig. 7c and Supplementary Fig. 6e).

### Complex collar structure

The collar of the tail consists of a complex of the connector, the upper part of the lower collar and the 12 tail appendage arms. We determined the collar complex structure to a resolution of 3.2 Å by using focused refinements and reconstructions (Supplementary Fig. 2c, Methods). The results showed that the expanded narrow end of the connector sits on the inner wall of the wide mouth of the lower collar (Fig. 8a, b). A short loop connecting β8 and α4 of the narrow end is adapted into a pocket between α3, α6 of one gp11 monomer and α4, α6 of a neighboring gp11 monomer (Fig. 8b). Residue Asp165 of the short loop forms a salt bridge with residue Arg62 of the loop between gp11α3–4. The C-terminal four residues of gp11 (Met290Leu291Val292Tyr293), which consists of three hydrophobic residues, are inserted into the outer hydrophobic surface pocket of the connector narrow end that was generated by the expansion of the narrow end (Fig. 8b and Supplementary Fig. 4b).

Our results showed that each tail appendage of a gp12* trimer can be roughly divided into three structural segments, including the N-terminus (gp12*-N, residues 1–32), the arm stem (residues 32–100) and the tassel (residues 101–691) (Fig. 8a, c). The arm stem is a left-handed three-helix coiled-coil. The N distal end of the coiled-coil stem in proximity to the collar has one helix at an upper position (gp12*-upper α) near the neck of the connector and the other two helices (gp12*-lowerL α and gp12*-lowerR α) at a lower position near the interface where the narrow end of the connector attaches to the lower collar. Binding of the stems to the collar occurs through extensive interactions of gp12*-Ns with both the lower collar and the connector. Unlike the coiled-coil arm stem, the three corresponding N termini of each appendage spike (N-upper, N-lower-L, N-lower-R) are well separated and extend in different directions (Fig. 8c and Supplementary Fig. 7a). Residues 22–31 of the N-upper fill the gap between two gp10-α1s at the neck region and have hydrophobic and hydrophilic interactions with the neck region of the connector. The density of the rest of N-upper is not visible, suggesting a disordered conformation. The 24 N termini at the lower positions form a unique interlocked assembly (Fig. 8a, c). The N-lower-L extends underneath the stem and interacts with both the gp12*-lowerR α and the upper outer surface of the lower collar. The distal end of the N-lower-L protrudes radially filling in the groove in between the gp12*-upper α and gp12*-lowerL α of the right side neighboring stem, and stops near where the stem bends (Supplementary Fig. 7a). N-lower-R extends to the right side neighboring stem, traverses the coiled-coil arm between the ends of gp12*-upper α and gp12*-lowerL α and has extensive interactions with the narrow end of the connector, gp12*-upper α of the neighboring stem and the C-terminus of gp11. The distal end of N-lower-R also protrudes radially for 57 Å filling in the groove between gp12*-upper α and gp12*-lowerR α of the neighboring stem (Fig. 8c and Supplementary Fig. 7a). The coiled-coil stems protrude radially from the collar for 72 Å (Fig. 8a). For most of the tail appendages, the coiled-coil stem then bends for ~90° and extends further down for ~15 Å to the tassel portion. Indeed, the extended portion of the arm stem is a three-helix coiled-coil glued with the distal segments of two gp12-N termini (N-lower-L and N-lower-R) from a neighboring appendage (Supplementary Fig. 7a).

Three-dimensional classifications focusing on the tassels showed that the appendages have two distinct distribution groups (groups 1 and 2) (Supplementary Figs. 1 and 7b). The distribution of the appendages in both groups show significant asymmetric features. The conformations of the group 1 appendages are consistent with previous observations^2,17^ with appendages 1 and 6 lifting “up” perpendicular to the tail axis. Group 2 appendages are all positioned in the “down” conformation in which the appendages are roughly parallel to the tail axis.

### Tail knob structure

Our reconstruction results of the tail knob showed that the six gp9 molecules are arranged as three dimers forming the cylindrical tube structure of the tail knob (Fig. 8d and Supplementary Fig. 7c, d), which is consistent with the reported crystal structure^24^. The 12-fold collar attaches the threefold tail knob at a fixed position and interacts with the gp9 tip β-barrel domain with a large buried surface (~648 Å^2^). Compared with the crystal structure of the knob^24^, small differences were observed in the tip β-barrel domain, which is a more compact assembly upon binding to the lower collar. In the mature phage, the L-loops located at the distal end of the tail knob form a helix bundle within the tail knob tube (Fig. 8d and Supplementary Fig. 7c). However, in the genome DNA emptied phage, the L-loops exit upon the ejection of the genome DNA and form an alpha helix barrel that has a hydrophobic outer surface and an inner channel of ~32 Å in diameter (Fig. 8d and Supplementary Fig. 7d, e). The densities of the residues at the distal end of the helix barrel are only visible when the map is low-pass filtered to a lower resolution of 15 Å (Supplementary Fig. 7d), indicating disordered structures, which could become ordered following insertion into the lipid membrane and formation of the channel. The height of the helix barrel and the disordered tip is ~37 Å, which is close to the thickness of a lipid bilayer (Fig. 8e).

## Discussion

**Fig. 9:**
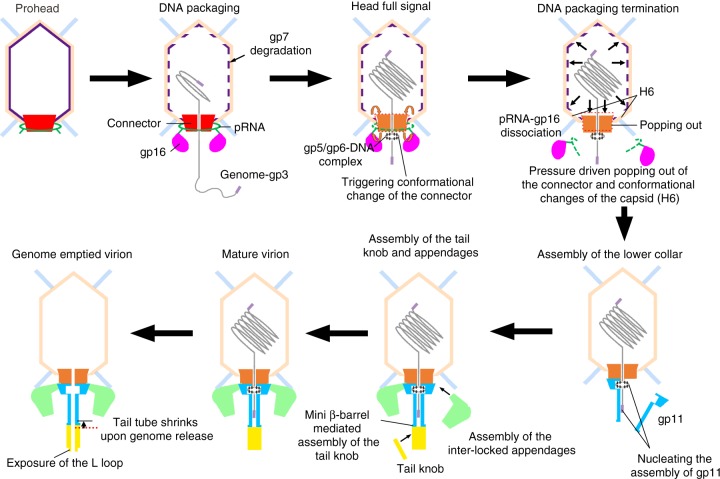
A schematic diagram showing the assembly, maturation, and infection of the bacteriophage ϕ29. Molecular details are illustrated for the dsDNA packaging, dsDNA packaging termination, assembly of the lower collar, tail knob and tail appendages, and genome ejection of the bacteriophage ϕ29

Our results show structural details of the complex capsid, the DNA packaging motor and the entire tail machinery, and provide a molecular basis for understanding the assembly, maturation and infection mechanisms of the bacteriophage ϕ29 (Fig. 9). The mechanisms revealed for the bacteriophage ϕ29 should be common for many other short non-contractile tail bacteriophages (Fig. 9).

Unlike the HK97 capsid in which the capsomeres are covalently crosslinked through inter-isopeptide bonds, the ϕ29 capsid gains increased stability through the interactions generated by the additional N and C-terminal domains. In addition, a large number of the salt bridges are formed between the capsid proteins (Supplementary Table 3). The head fibers at the quasi-3-fold positions can further stabilize the capsid. Previous studies have shown that the scaffolding protein is the determinant of the ϕ29 capsid form. It has been estimated that there are ~150–180 scaffolding molecules in each prohead^6,33,34^. The total number of scaffolding molecules underneath the pentameric capsomeres are 110, thus the rest 40–70 scaffolding molecules could form the weak concentric density shell underneath the capsid, as suggested in previous studies^14^.

For many bacteriophages such as HK97, T4 and P22, the capsids undergo dramatic conformational changes during maturation and the scaffolding protein might exit through holes in the capsid during capsid maturation^35–37^. The ϕ29 capsid undergoes only miniscule conformational changes upon genome loading and release and does not generate sufficiently large holes for the exit of the scaffolding protein (Supplementary Fig. 3d, Supplementary Table 4). Upon the genome packaging, the central channel of the connector, which is the only channel that is sufficiently large for passage of the scaffolding protein, is blocked by dsDNA. Thus, it is unlikely that the scaffolding proteins can exit from the head during or after genome packaging. Mass spectrometric analysis of the mature phage showed peptide fragments of the scaffolding proteins, suggesting that the scaffolding proteins may be degraded into small fragments (Supplementary Fig. 6a and Supplementary Table 5) as having been observed for other phages^38,39^.

Based on our near-atomic resolution structures of the pRNAs, it can be confirmed that there are five pRNA molecules in the ring-shaped RNA structure. Since the pRNA ring is unable to adapt to the expanded connector, it is reasonable to deduce that expansion of the connector narrow end triggers the disassembly of the pRNA and DNA packaging motor. In addition, we did not observe any pRNA density in the 3.2 Å mature capsid map. The conformational change of the E loop, although only corresponding to an ~3 Å shift, could completely abolish the binding of the pRNA to the capsid (Figs. 6b, 9).

It was proposed that the connector might rotate during the packaging of the genome^40^. However, later experimental data suggested no free rotation of the connector during this process^41^. Instead, recent studies have shown that the genome rotates during packaging into the head^42–45^. Our results suggest that oscillation or local rotation within a limited region (<6 degrees) is still highly possible during genome packaging or release (Fig. 5c and Supplementary Fig. 2e). In addition, connector rotation must occur during the maturation of the capsid.

Observations of the positional and conformational changes of the connector suggest a new mechanism in sensing the head full, in which both the internal pressure at the final stage of the genome packaging and the triggering of the connector conformation change are critical (Fig. 9). It has been reported that the in vitro packaging system of ϕ29 can package 10% more of the genome^10^. Although the differences between the in vitro and in vivo conditions may lead to differences in the internal pressure generated that may in turn affect the total amount of packed DNA^46^, the pressure alone cannot precisely control the length of packed DNA. The toroid protein–genome complex at the distal end of the genome may trigger the initial conformational change in the narrow end of the prohead connector and play a key role in the precise control of packing only one genome. Conformational changes in the narrow end could then be transmitted to induce conformational changes of the capsid and the wide end of the connector. In the presence of high internal pressure (~100 atm)^47^, the connector and the capsid undergo conformational transition and the transformed connector is popped out by the pressure (Fig. 9). However, for bacteriophages that do not precisely controlling the length of packed DNA, the internal pressure could serve as the only signal for head full by pushing the connector to a disabled position or triggering the conformational changes in the connector to a disabled state that terminates the DNA packaging^3^.

Recombinant gp11 does not self-assemble into lower collar structures (Supplementary Fig. 8), suggesting that assembly of the lower collar requires other factors. Both 5ʹ ends of the genome are covalently attached to the terminal protein gp3. Gp3 should be the last piece remaining outside the capsid at the final stage of the genome packaging. It is possible that gp3 functions as a nucleus for the assembly of the lower collar (Fig. 9). The expanded narrow end of the connector upon head full is also expected to be involved in the lower collar assembly since gp11 is unable to form a complex with the isolated gp3-genome (Supplementary Fig. 8b). The interlocked assembly of the appendage suggests that signals sensed by one tail appendage could be transmitted to and sensed by other tail appendages, revealing a unique mechanism in host-cell recognition and probably in triggering dsDNA injection upon the irreversible anchoring on the host-cell surface^21^.

The DNA packaging motor packages the ϕ29 genome to near-crystalline densities and the internal pressure that builds up in the capsid at the final stage of DNA packaging can reach as high as ~100 atm^10,47^. Previous mutagenesis and recent simulation studies have suggested that the connector functions as a “Chinese finger trap” check valve preventing pre-mature DNA leakage^41^. However, the connector can only temporarily hold the packaged DNA as demonstrated by the minus-gp11 mutant phage, which loses its packaged DNA shortly after it is isolated^20^. Thus, the tail plays an essential role in stably holding the DNA, most likely through electrostatic interactions between the terminal protein gp3 and the tail tube. The release of the genome triggered by low-pH buffers in vitro could be the result of disruption of the electrostatic interactions between gp3 and the tail tube and induction of the tail tube conformational changes, which disturbs the packaged genome. However, such an extreme low-pH environment may not exist on the bacterial surface during the infection. Thus, the mechanisms involved in the triggering of genome injection in vivo remain to be fully elucidated.

## Methods

### Phage production

Fibered ϕ29 proheads were produced in the non-permissive *Bacillus subtilis spoA12* cells infected with *sus*16(300)-*sus*14(1241) phage as previously described^16^. Briefly, the cells were grown in 50 ml 416 media (2% (w/v) Difco Bacto-tryptone, 1% (w/v) Difco yeast extract, and 0.17 M NaCl) to an OD_600_ value of ~1.2 (~4 × 10^8^ cells/ml). The cells were pelleted by centrifugation at 2000 × *g* and were then resuspended in 10 ml fresh 416. Mutant *sus*16(300)-*sus*14(1241) phage were added to the cells at a M.O.I. of 15. After 5 min at 37 °C, the infected cells were transferred to 90 ml pre-warmed fresh 416 medium and were cultured at 37 °C; in a shaking incubator for ~70 min The cells were harvested by centrifugation at 10,000 × *g* and were then resuspended in 1 ml RNase free PBS buffer with 10 μg/ml lysozyme (Sigma) and 10 μg/ml DNase I (Calbiochem). The cell lysate was clarified by centrifugation, and the proheads were purified using a 17–30% linear sucrose density gradient in TMS buffer (containing 100 mM NaCl, 10 mM MgCl_2_, and 50 mM Tris at pH 7.8).

Fibered mature phages were produced in the permissive *Bacillus subtilis su44*+ cells infected with *sus*16(300)-*sus*14(1241) phage. The procedure is similar to that for the prohead production. Approximately 2.5 h after infection, most of the cells were lysed by the phage. Lysozyme (at a final concentration of 20 μg/ml) and DNase I (at a final concentration of 2 μg/ml) were added to the media for complete lysis of the cells. The cell debris was removed by centrifugation at 10,000 × *g*. Mature phage particles were then collected by centrifugation at 20,000 × *g* and were resuspended in TMS buffer, and then were purified in an isopycnic 65% (w/v) CsCl gradient.

The genome emptied ϕ29 particles were produced by using a previously described procedure^24^. Briefly, the purified fibered mature ϕ29 phages in a buffer containing 50 mM Tris 100 mM sodium chloride and 10 mM magnesium chloride were adjusted to a pH of ~4.2 with 0.1  M sodium acetate at pH 4.0, 300 mM ammonium sulfate and 0.8% (v/v) Triton X-100. After being incubated at 37 °C for 20 h, the low-pH-treated particles were purified in an isopycnic 65% (w/v) CsCl gradient with 0.02% (v/v) Triton X-100.

### CryoEM data collection and image processing

The prohead samples were vitrified over ultrathin lacey carbon grids (Ted Pella, INC.) using a Gatan Cryoplunge 3 system and were imaged at a nominal magnification of 59,000 on Kodak SO-163 films in an FEI Titan Krios microscope operating at 300 kV. An electron dosage of ~25 e/Å^2^ was used for each film. Micrographs were digitized with a Nikon Coolscan 9000ED scanner at 6.35 μm intervals, which yielded an effective pixel size of 1.1 Å. A total of 527 micrographs exhibiting no significant astigmatism, drift or charging problems, and defocus value of 1.4–2.8 μm were selected for further processing.

Mature phage samples were vitrified over 400 mesh Quantifoil grids (1.2 μm hole size) using the Gatan Cryoplunge 3 system. CryoEM data of the mature phage were collected as movie frame stacks on a K2 Summit direct electron counting camera (Gatan Inc.) with a nominal magnification of 22,500 (an effective pixel size of 1.32 Å) in an FEI Titan Krios operating at 300 kV. Each stack has 32 image frames and the accumulated electron dosage for the entire stack is ~ 40 e/Å^2^. UCSFImage4 was used for all data collection (developed by X.L.). The movie frames of each stack were aligned and summed up into a single image using the program motioncorr^48^ before subsequent processing. The anisotropic magnification distortion parameters of K2 Summit direct camera were estimated as previously described^49^. In brief, 28 movie stacks of polycrystalline gold were collected at the super-resolution mode (an effective pixel size of 0.66 Å) under the same magnification as that for the mature phage data collection. The stacks were then processed for motion correction. Motion-corrected images of each stack were summed up and the single sum-up image was binned by a factor of 2 × 2, which yielded a pixel size of 1.32 Å. Then the distortion parameters were estimated for each single sum-up image using the program mag_distortion_estimate^49^. The average distortion angle determined from 28 single sum-up images is 137.4 degree and the average scale for the long axis determined is 1.019. The pixel size after distortion correction is 1.30 Å. The motion-corrected single images of the mature phage were corrected for anisotropic magnification distortion using the program mag_distortion_correct with the parameters determined using the polycrystalline gold^49^. A total of 1899 corrected images without significant astigmatism, drift or charging problems and defocus value of 1.0–3.3 μm were selected for further processing.

Preparation of the cryo-EM grids of the genome emptied virions was the same as that for the prohead. The cryo-EM data of the genome emptied virions were collected as movie stacks on a Falcon II camera with a nominal magnification of 59,000 (an effective pixel size of 1.35 Å) in an FEI Titan Krios operating at 300 kV. Each stack contains 19 frames and the accumulated dose rate in each stack was ~50 e/Å^2^. The collected datasets were processed for motion correction at micrograph level using the program dosefgpu_driftcorr. A total of 2597 corrected images without significant astigmatism, drift or charging problems and defocus value of 1.0–2.5 μm were selected for further processing. Representative raw micrographs of different particles are shown in Supplementary Fig. 9a.

### CryoEM structure determination

A total of 45,577 prohead particles, 54,741 mature virion particles, and 48,437 genome emptied virion particles were manually boxed with the EMAN2 program e2boxer.py^50^. The python script *fitctf.py*^27^ was used to determine the defocus value and the astigmatism parameters for each micrograph. The boxed particles were subjected for two-dimensional classifications using the program Relion^51^. Particles in classes with clear structure features were selected, combined, and used for further processing. The selected and combined particles were randomly split into two halves and were processed separately till the final cycle of the refinement^52^. For the prohead reconstruction, an initial model was generated by scaling and low-pass filtering a previously determined low-resolution prohead map to a resolution of 40 Å^16^. For the reconstructions of the mature head and the genome emptied head, initial models were generated by scaling and low-pass filtering a previous determined mature head map to a resolution of 40 Å^2^. Initial reconstructions of the heads were calculated against images binned by a factor of 2 × 2 using the program EMAN assuming fivefold symmetry. Orientations and centers of each particle determined by EMAN were further refined by a fine local search against unbinned images with the python script jspr.py^53^. Defocus values of individual particles were refined at the late stage of the refinement with the python script jspr.py^53^. The two maps reconstructed from the randomly split two halves were aligned after each iteration of refinement and were then used as the initial models of the corresponding half datasets for the next iteration of refinement. Convergence of the refinements and the resolutions of the reconstructions were estimated by the Fourier shell correlation (FSC) curves calculated between the randomly split two halves. The final map was calculated from all the data. Total 18,230 prohead particles, 36,730 mature virion particles, and 44,059 genome emptied virion particles were used in the final reconstructions assuming fivefold symmetry. Structure of the ϕ29 prohead, the mature virion head and the genome emptied virion head was calculated assuming fivefold symmetry to a resolution of 3.6, 3.2, and 3.2 Å, respectively, (Supplementary Fig. 2a). The quality of the maps was visual checked and compared with density maps at similar resolutions, the results showing consistent features with the corresponding resolution obtained. The final maps are good enough for ab initio model building of the capsid shell. Atomic models were constructed for 47 copies of the major capsid protein gp8 (residues 4–438/448) and 33 copies of the minor capsid protein gp8.5 N-terminal domain (residues 1–116) in the asymmetric unit of the head. Densities of the C-terminal ten residues in some of the gp8 subunits are not visible. The C-terminal residues 117–280 of gp8.5 form the fibrous part of the head fibers. Densities for the gp8.5 C-terminal domain are weak and show only low-resolution structural features, indicating possible vibrations of the C-terminal fibrous part. The crystal structure of the gp8.5 C-terminal domain (residues 118–280, PDB entry: 3QC7, 10.2210/pdb3QC7/pdb)^19^ is fitted into the cryoEM density as a rigid-body for building a complete atomic model of the head fiber. Symmetry related capsid proteins were generated with the symmetry operators and then were merged to give a complete model for each head. The whole atomic models were refined against corresponding density maps using the PHENIX real space refinement and RosettaCM^54,55^ (Supplementary Table 1). The C-terminal domains of gp8.5 were fixed and refined as a rigid-body during all the refinements.

The densities for the pRNA molecules are weak in the fivefold averaged 3.6 Å prohead map. Further three-dimensional (3D) classification of the prohead focusing on the pRNAs using Relion showed four distinct classes with a ~34% of particles in class 1, 12% of particles in class 2, 18% of particles in class3 and 36% of particles in class4 (Supplementary Fig. 1). Class1 shows a clear ring of pRNA density in which the structural features of the RNA duplexes could be clearly identified (Supplementary Figs. 1 and 5a). Class2 shows no pRNA density. Class3 shows features in consistence with the base of the head fibers, suggesting that the orientations of the particles were incorrectly determined as the 180º-inverted pseudo C2 equivalent. Class4 shows five featureless and disconnected blobs, which might represent disordered pRNAs. Particles in difference classes were subject for focused refinements. Only particles in class1 yield an interpretable map and the resolution measured was 4.6 Å (Supplementary Figs. 1 and 5a). A pentameric atomic model of five pRNA molecules (each consists of 5ʹ 69 nts from 26–94) was built based on the reconstruction from the particles in class 1 and the crystal structure of a 71 nt fragment of the pRNA (PDB entry: 3R4F, 10.2210/pdb3R4F/pdb)^56^. The model was refined against the density map with the program ERRASER of the Phenix package^57^ and then the coordinates were merged into the atomic model of the prohead. For classes 2 and 4, focused refinements were also performed on the pRNAs by systematic orientation search around the long axis of the head and with a sixfold symmetry imposed. However, none of the reconstructions produced interpretable pRNA densities.

Because of the symmetry mismatch between the head and the tail/connector, densities of the tail/connector are smeared in the fivefold averaged head reconstruction. Assuming a fixed relationship between the head and the tail/connector, orientations of the connector/tail were determined by searching the five equivalent positions derived from the corresponding head orientation^2,4,58^. However, a reasonable reconstruction can only be obtained for the mature virions. A 5.9 Å map of the entire mature virion was calculated without assuming any symmetry (asymmetric reconstruction) (Supplementary Fig. 1). Alternatively, reasonable results can be obtained for the connector of the prohead and the tail of the genome emptied virion, through a systematic orientation search around the symmetry axis. Further analysis of the orientations confirmed the none-fixed relationship between the head and the prohead connector/the tail of the genome emptied virion (Supplementary Fig. 2e). A 3.8 Å map of the prohead connector and 3.6 Å map of genome emptied virion connector were calculated assuming 12-fold symmetry with the images in which the capsid density was subtracted (Supplementary Figs. 1 and 2b). The atomic model of the prohead connector was built by using the connector crystal structure as a reference (PDB entry: 1FOU, 10.2210/pdb1FOU/pdb) and was then refined against the EM map. The final model contains residues 12–228 and residues 247–285 of each gp10 molecule. The atomic model of the mature virion connector was built in COOT and was then refined against the EM map. The final model contains residues 7–229 and residues 245–285 of each gp10. The atomic model of the genome emptied virion connector was also built in COOT and was then refined against the EM map. The final model contains residues 7–229 and residues 245–301 of each gp10. The C termini (residues 286–301) of the gp10s are visible only in the genome emptied virion connector structure and were built as alanines. The tail is about 360 Å long. We found that it was difficult to obtain a high-resolution reconstruction of the entire tail either for the mature or the genome emptied virion, probably due to the slight bend in the tail. In addition, the tail knob and the tail axis have different symmetries (3-fold vs. 12-fold). Thus, the tail was split into three segments, including the collar complex, the tail axis and the tail knob (Supplementary Fig. 1), for focused refinements, in which a small local mask was used and the centers of the boxed images were moved from the head to each corresponding segment. At the final stage of the refinements, local searches in the other two dimensions were also allowed in a limited region of <5 degree. The structure of the collar complex, which includes the connector, the upper part of the lower collar and the tail spike arms, was determined assuming C12 symmetry to a resolution of 3.3 Å for the mature virion and 4.0 Å for the genome emptied virion (Supplementary Fig. 2c). The structure of the lower collar was determined assuming C12 symmetry to a resolution of 3.3 Å for the mature virion and 3.8 Å for the emptied virion. The structure of the tail knob was determined assuming C3 symmetry to a resolution of 6.7 Å for the mature virion and 4.2 Å for the emptied virion (Supplementary Fig. 2d). Atomic models for the connector dodecamers, the N-terminal arms of the tail spikes, and the low collar gp11 dodecamers were built and refined against corresponding EM maps. Since the density of link region between tail appendage arm stem and tassel part could only be visible after low-pass filtered to 5 Å, the C distal end of arm stem in gp12^*^ were built as poly-alanine peptide chain. For the lower collar assembly of the genome emptied virion, residues 170–192 of gp11 were built as alanines since the density is not good enough for side chain assignment. Atomic models of the tail knobs in the mature and the genome emptied virion were built by fitting the crystal structure (PDB entry: 5FB5, 10.2210/pdb5FB5/pdb)^24^ into the cryoEM maps. The alpha helix barrel at the distal end of the tail knob of the genome emptied virion was built as Cα atoms in COOT. Residues 440–455 of gp9 are disordered in either the mature tail knob structure or the genome emptied tail knob structure. The tail appendages in “up” and “down” conformations are highly flexible and do not have recognizable features. Further 3D classifications of the particles focused on the tail spikes resulted in two major groups with one group having spikes at positions 1 and 6 adopting the “up” conformation, whereas the other group having all the spikes in the “down” conformation (Supplementary Figs. 1 and  7b). Trimeric features of the spikes are clearly visible in both groups. Pseudo atomic models of the spikes in the two groups were built by fitting the crystal structure of the spike trimer (PDB entry: 3GQ7, 10.2210/pdb3GQ7/pdb) into the cryoEM densities. The final models of the tail appendages contain residues 21–691 of the upper gp12*s, residues 14–691 of the lower-L gp12*s and residues 3–691 of the lower-R gp12*s. Gp13, the lysozyme-like tail protein of ϕ29, was not identified in the reconstructed densities. Atomic models for the entire prohead, the mature virion and the genome emptied virion were built by merging relevant atomic structures with the corresponding EM map as a reference. Atomic models of different subgroups were also built using a similar method.

Additional densities are visible underneath the pentameric capsomeres of the prohead. However, the densities are not strong enough for interpretation. The densities were extracted and were averaged using the local fivefold symmetry with the Uppsala software ave^59^. The averaged densities showed clear features consistent with the arrow shaped scaffolding dimer. The densities were interpreted by fitting the crystal structure of the scaffolding protein structure (PDB entry: 1NOH, 10.2210/pdb1NOH/pdb)^14^ into the density map using Chimera^60^.

### Crystal structure determination of the gp8.5 N-terminal domain

The full-length gp8.5 consists of 280 residues. Previous crystal and cryoEM structural analysis of the gp8.5 C-terminal part show that the C-terminal ~170 amino acids forms the fibrous portion of the head fiber, leaving the N-terminal ~110 amino acids forming the base of the head fiber. A gene construct containing residues 1–117 of gp8.5 was PCR amplified from the genomic DNA of bacteriophage ϕ29 using the following primers: 5ʹ-CCCATGGGCAATGACTATCTATCTGC-3ʹ and 5ʹ-CCGCTCGAGTCAGTGGTGGTGGTGGTGGTGCCTCAATTCATTCTCGACGC-3ʹ. The purified PCR products were cloned into pET28b (Novagen) using *Nco*I-*Xho*I sites that placed a His_6_ tag on the C-terminus of the recombinant protein. The recombinant gp8.5 N-terminal domain was expressed in *E. coli* BL21(DE3) RIL cells at 20 °C. The recombinant protein was affinity purified using cobalt-charged BD TALON^TM^ resins. The protein eluted from the cobalt beads was concentrated and further purified with a Superdex 200 column (GE) in a buffer containing 20 mM Tris at pH 8.0 and 100 mM sodium chloride. The purified protein was then concentrated to ~10 mg/ml for crystal screening. Initial crystal screenings were set at 20 °C using the Hampton screening kits Crystal Screen I and II. Optimization of the crystallization conditions was performed by hanging-drop vapor diffusion in 24-well plates using 2 μl of protein (10 mg/ml) mixed with an equal volume of well solution. Diffraction quality crystals of the gp8.5 N-terminal domain were grown in 0.1 M HEPES at pH 6.0, 0.15 M ammonium sulfate and 4% v/v DMSO. Crystals were soaked for 10 s in the well solution containing a final concentration of 20% v/v glycerol to flash freeze in liquid N2.

X-ray diffraction data of the gp8.5-N base crystals were collected using synchrotron radiation at the Advanced B beamline 23D and the Shanghai Synchrotron Research Facility beam line BL17U (Table 1). The best crystal of the gp8.5-N base diffracted to 1.8 Å and belonged to space group *P2*_*1*_, with six molecules in the asymmetric unit and cell parameters of *a* = 55.2 Å, *b* = 65.1 Å, *c* = 118.9 Å, *α* = 90°, *β* = 99.091°, *γ* = 90°. The data were integrated and scaled with the HKL2000 suite (Table 1)^61^.

The structure of the gp8.5-N base was determined by molecular replacement with Phaser using the EM map as a search model. The program COOT was used for model building and for making adjustments^62^. The program PHENIX was used to refine the structure (Supplementary Fig. 9b and Table 1)^54^. The gp8.5-N base structure was refined to a Rwork/Rfree of 0.182/0.224. Of all the residues, 97.0% are in the most favored regions of the Ramachandran plot and none of the residues are in the disallowed regions.

### Relative orientation of the connectors

To calculate relative orientation of the connector to the corresponding head, the 12 equivalent orientations of the connector were generated using the symmetry operators. Then pair-wised comparisons were made between each equivalent orientation of the connector and orientation of the corresponding head. The minimal absolute differences in azimuth^63^ were used for final plot and analysis. (Supplementary Fig. 2e) The differences in alt and phi were used to exclude outliers. Particles were discarded for those that the angular differences in alt or phi are >5 degrees.

### Magnification calibration

Since the cryoEM data of the prohead, the mature virion and the genome emptied virion were collected from different electron microscopes using different detectors or film, the magnification of each reconstruction was carefully calibrated using crystal structures as references. The crystal structure of the gp8.5 N-terminal domain was served as a reference for the magnification calibration of the mature virion and the genome emptied virion reconstructions. Briefly, the EM density map of one gp8.5 N-terminal domain was extracted from the corresponding reconstruction. A series of pixel values were then applied to the map using e2proc3d.py to generate maps with different pixel values. Each of these maps was then converted to *hkl*s by using Sfall of the CCP4 package^64^. Position of the molecule in the map was searched in reciprocal space with the crystal structure as a rigid-body search model using the program MolRep. Fourier space correlation coefficients (CC) of the best search results were collected and used for a CC-pixel plot. The calibrated pixel value, correlated to the maximum CC value, was estimated from the fitting curve of the plot. The calibrated pixel values of the mature virion reconstructions and the genome emptied virion reconstructions were 1.295 Å/pixel and 1.360 Å/pixel, respectively (Supplementary Fig. 9c).

The quality of the prohead gp8.5 N-terminal domain density was not as good as those of the mature virion and the genome emptied virion reconstructions. The crystal structure of the connector (PDB:1FOU, 10.2210/pdb1FOU/pdb) was used for the magnification calibration of the prohead reconstructions. The final calibrated pixel value of the prohead was 1.072 Å/pixel (Supplementary Fig. 9c).

### Expression, purification, and characterization of gp11

The full-length of gp11 protein comprises of 293 residues. The gp11 gene was PCR amplified from the genomic DNA of bacteriophage ϕ29 using the following primers: 5ʹ-GAAGGAGATATACATATGAGTAGTTACACAATGCAGTTA-3ʹ and 5ʹ-TGGTGGTGGTGCTCGAGTTAATGATGATGATGATGATGATACACAAGCATAAACAGTTC-3ʹ. A His_6_ tag was introduced to the C-terminus of the recombinant gp11 through the PCR primers. The PCR product was cloned into pET30b (Novagen) digested with *Nde*I and *Xho*I using the infusion method (Clontech). The recombinant gp11 was expressed in *E.coli* BL21(DE3) cells at 16 °C for 20 h following the standard protocol for IPTG-induced protein expression in *E. coli* cells (see the Novagen pET system handbook). The produced recombinant protein was affinity purified using cobalt-charged BD TALON^TM^ resins and eluted from the cobalt beads using an elution buffer containing 20 mM HEPES, 150 mM sodium chloride and 500 mM imidazole. The eluted protein was concentrated and further purified with a Superdex 200 column (GE) running in a buffer containing 20 mM HEPES 7.5, 150 mM sodium chloride. The protein samples from different peaks or peak fractions mixed with dsDNA or the gp3-genome were checked by using negative staining electron microscopy.

### Analysis of the ssDNA–gp5–gp6 complex

The gp5 gene was PCR amplified from the ϕ29 genome DNA with the primers: 5ʹ-GAAGGAGATATACATATGCATCATCATCATCATCATATGGAAAACACAAACATCGTAA-3ʹ and 5ʹ-TGGTGGTGGTGCTCGAGTTATAGGGATAGTTGTAAGCTAAAG-3ʹ. The gp6 gene was PCR amplified from the ϕ29 genome DNA with the primers: 5ʹ-GAAGGAGATATACATATGGCAAAAATGATGCAGAGAGAAATC-3ʹ and 5ʹ-TGGTGGTGGTGCTCGAGTCAATGATGATGATGATGATGTTCAGCAACCTGTTCTTCTGG-3ʹ. The gp5 and gp6 genes were cloned, respectively, into the plasmid pET30b digested with *Nde*I and *Xho*I by infusion. His_6_ tags were introduced through PCR primers to the N-terminus of gp5 and the C-terminus of gp6. The recombinant gp5 and gp6 were expressed in *E.coli* BL21(DE3) cells at 16 °C for 20 h. Expression of the recombinant proteins was induced by adding 1 mM IPTG. The produced recombinant proteins were affinity purified using cobalt-charged BD TALON^TM^ resins and eluted from the cobalt beads using an elution buffer containing 10 mM HEPES and 500 mM imidazole. The DNA contaminations in imidazole elution were removed with a HitrpQ column. The protein fractions eluted from the HitrpQ column were concentrated and further purified with a Superdex 75 column (GE) running in a buffer containing 10 mM HEPES 7.5. The purified gp5 and gp6 proteins were mixed with two desthio-biotin labeled single-stranded DNA fragments, of which the sequences are the same as the forward and reverse strands of the right end 21–79 bp of the ϕ29 genome (forward-5ʹ- CATACACCATTTCCCCATTGACCGACTATCTTCGACAAGAATCTAACAACTAAATCACG-3ʹ, reverse-5ʹ-GTATGTGGTAAAGGGGTAACTGGCTGATAGAAGCTGTTCTTAGATTGTTGATTTAGTGC-3ʹ). Magnesium chloride was added to the mixture at a final concentration of 10 mM. The mixture was incubated at 37 °C for 30 min and was then successively affinity purified with Strep and Cobalt resins. The eluted sample was used for SDS-PAGE and negative staining analysis.

### Quantification and statistical analysis

All reported resolutions are based on the gold-standard Fourier Shell Correlation (FSC) = 0.143 criteria^65^.

### Reporting summary

Further information on research design is available in the Nature Research Reporting Summary linked to this article.

## Supplementary information

Supplementary Information

Reporting Summary

## Data Availability

The atomic coordinates, structure factor files, and EM maps have been deposited into the Protein Data Bank and the EM Data Bank with the following accession numbers: EMD4655, 6QVK (prohead: head), EMD4662, 6QX7 (prohead: connector), EMD4680, 6QYZ (prohead: pRNA), EMD4677, 6QYD (mature virion: head), EMD4678, 6QYJ (mature virion: connector), EMD4681, 6QZ0 (genome emptied virion: head), EMD4679, 6QYM (genome emptied virion: connector), EMD4682 (mature virion: tail knob), EMD4683 (genome emptied virion: tail knob), 6QYY (gp8.5 N domain, crystal structure), EMD4684, 6QZ9 (mature virion: collar complex) and EMD4685, 6QZF (genome emptied virion: collar complex).
