# Supplementary material for: Structural assembly of the tailed bacteriophage ϕ29
Source: Nat Commun. 2019 May 30;10:2366. doi: 10.1038/s41467-019-10272-3 (PMC6542822; doi:10.1038/s41467-019-10272-3)
Supplement: Supplementary file 1 — Supplementary Information [file 41467_2019_10272_MOESM1_ESM.pdf]

Supplementary information for

# Structural assembly of the tailed bacteriophage ϕ29

Jingwei Xu[1, 2], Dianhong Wang[1], Miao Gui[1, 3], Ye Xiang[1*]

[1]Beijing Advanced Innovation Center for Structural Biology, Beijing Frontier Research Center for Biological Structure, Collaborative Innovation Center for Diagnosis and Treatment of Infectious Diseases, Center for Infectious Disease Research, Department of Basic Medical Sciences, School of Medicine, Tsinghua University, Beijing 100084

[2]Present address: Institute of Molecular Biology and Biophysics, Eidgenössische Technische Hochschule Zürich, CH-8093 Zürich, Switzerland

[3]Present address: Department of Biological Chemistry and Molecular Pharmacology, Harvard Medical School, Boston, MA 02115, USA

[*] To whom correspondence should be addressed: Y.X.: Tel.:+86-10-62772587; Email: yxiang@mail.tsinghua.edu.cn.

## Supplementary Figure 1

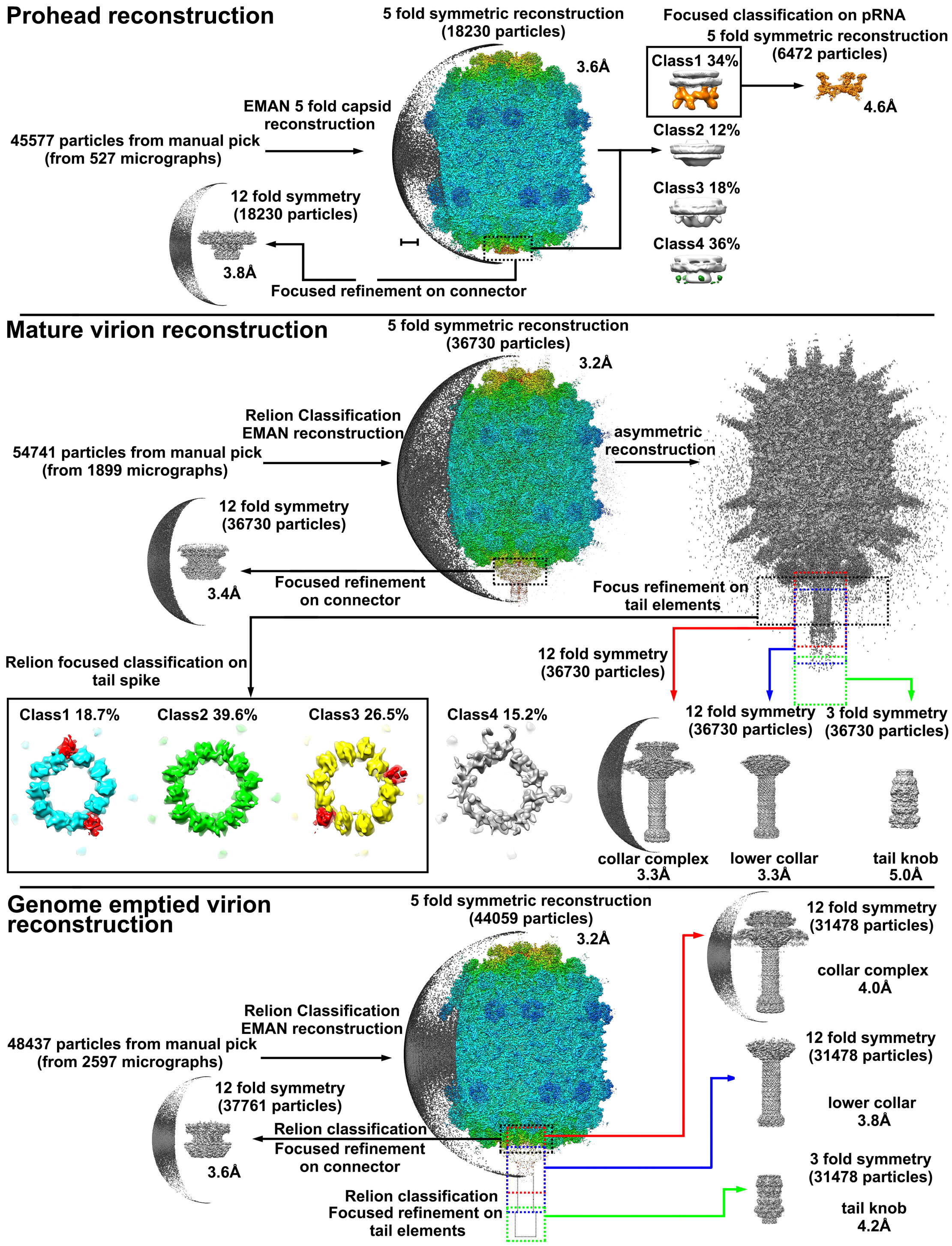

**Supplementary Figure 1. Workflow for structure determination**.

Flowcharts for the cryoEM 3D reconstructions of the bacteriophage ϕ29 prohead, the mature virion and the genome-emptied virion. Please refer to Methods and Supplementary Table 1 for details. The scale bar represents 5 nm.

## Supplementary Figure 2

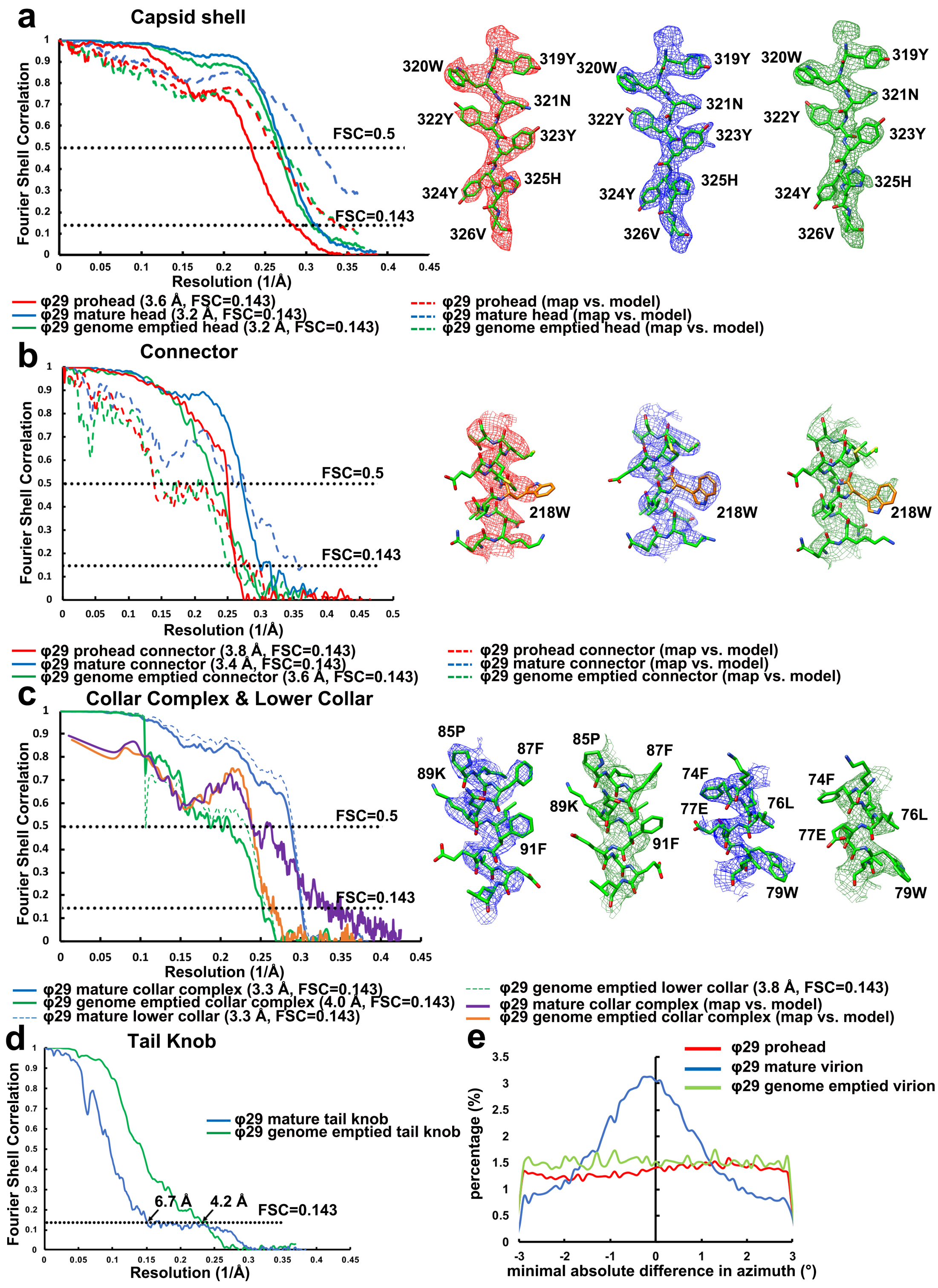

**Supplementary Figure 2. Cryo-EM analysis and representative EM density maps of different structure components.**

(a) Left: Gold standard Fourier shell correlation (FSC) curves of the prohead, the mature virion head and the genome emptied virion head reconstructions. Right: representative EM density maps.

(b) Left: Gold standard Fourier shell correlation (FSC) curves of the prohead connector, the mature virion connector and the genome emptied virion connector reconstructions. Right: representative EM density maps.

(c) Left: Gold standard Fourier shell correlation (FSC) curves of the collar complex and the lower collar reconstructions. Right: representative EM density.

(d) Gold standard Fourier shell correlation (FSC) curves of the tail knob reconstructions.

(e) The relative orientation of the connector comparing to the orientation of the corresponding capsid shell.

Supplementary Figure 3

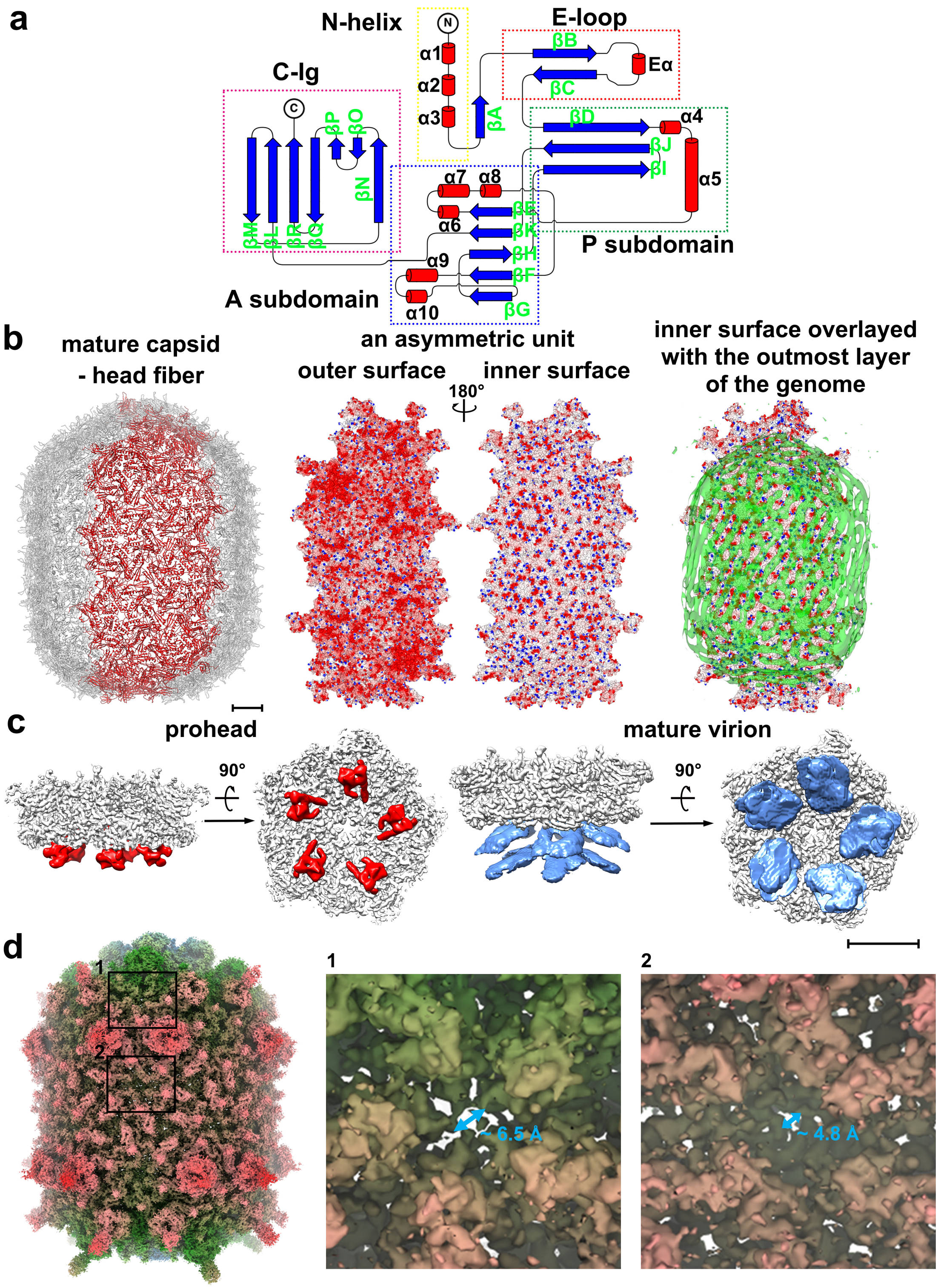

**Supplementary Figure 3. Diagrams showing the topology of gp8, surface electrostatic potential of the capsid, densities underneath the pentameric capsomeres and holes on the capsid.**

(A) Diagrams showing the topology of the capsid protein gp8. Secondary structures in the same subdomain are grouped in a frame. The domain and subdomains are colored the same as in "Figure 2a".

(B) Left: ribbon diagrams showing the fiberless mature capsid shell with one asymmetric unit colored orange; middle: outer and inner surface electrostatic potentials of the mature capsid. Negative and positive electrostatic potentials are colored red and blue, respectively; right: outmost layer of the genome merged with inner surface electrostatic potential of the mature capsid. The genome in association with equatorial capsomeres is organized in the oblique line. The positions of dsDNA complement positive charges on the inner surface of the capsid well. The scale bar is 5 nm.

(C) Side and upward views of the averaged densities underneath the pentameric capsomeres from the prohead and the mature virion head. The averaged density in the prohead (red) shows clear features consistent with the arrow-shaped scaffolding dimer. However, the averaged density in the mature virion head does not have any recognizable features (blue).

(D) Surface shadowed diagrams showing the holes on the capsid. The EM map of the mature virus head is set to a contouring level of 2.5 σ under which most side chains of the major capsid protein are visible. The map voxels are colored from green to red according to the distances between the map voxels and the 5 fold axis. The biggest holes on the capsid have a diameter of approximately 6.5 Å and are located at the centers of the pentameric capsomeres.

## Supplementary Figure 4

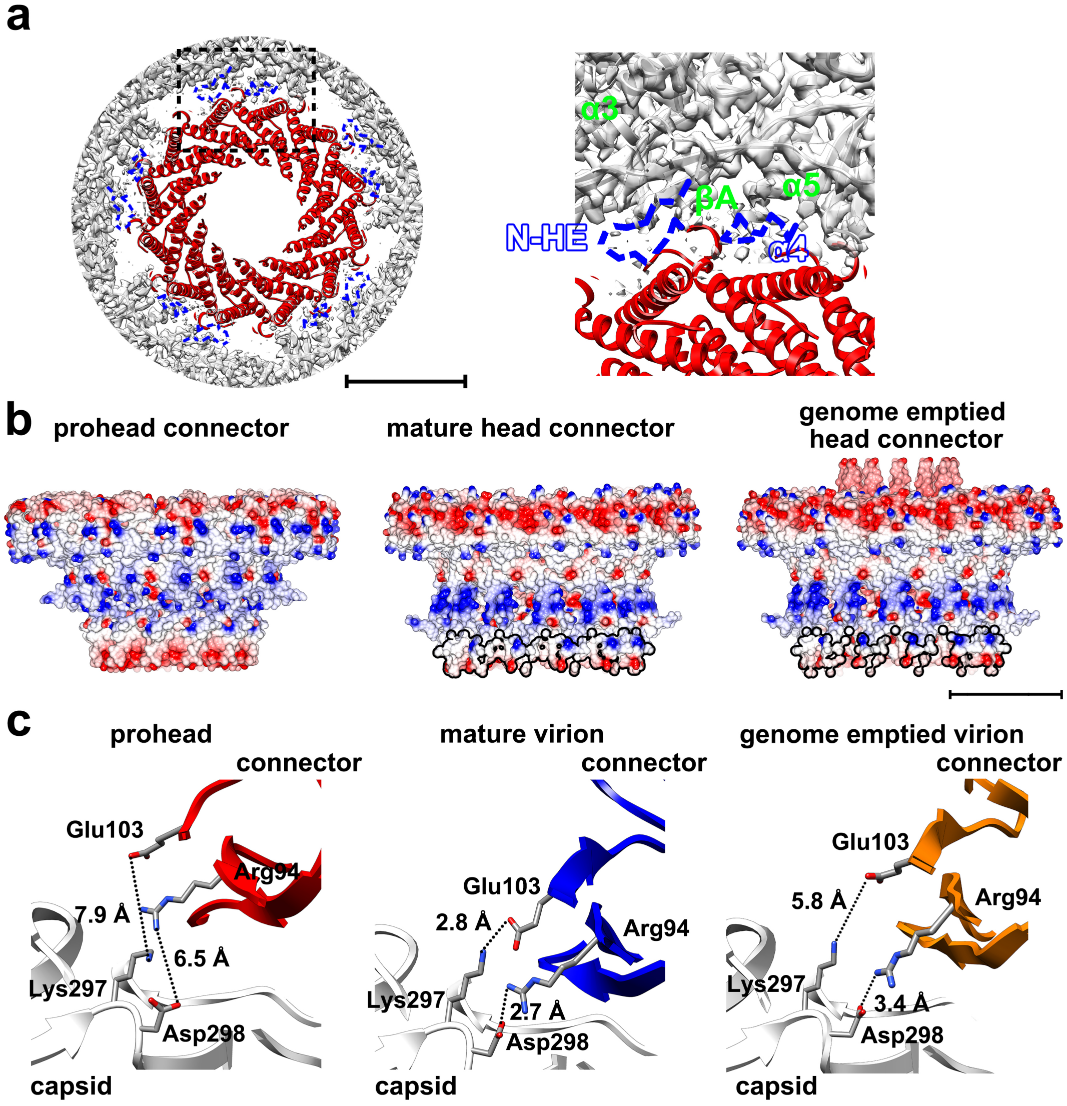

**Supplementary Figure 4. Structure of the connector.**

(a) Left: EM density around the interface of the capsid shell and the connector neck region. The connector is shown in ribbon diagrams and colored red. The disordered structures (gp8 N-arms and gp8-α4s of the five radially oriented HK97-P subdomains) are outlined with dashed blue lines; Right: a zoom-in showing the details of the disordered regions. Ribbon diagrams of the ordered capsid structure are shown in the density map. Secondary structures near the disordered regions are labeled. The scale bar is 5 nm.

(b) Surface electrostatic potential of the prohead, the mature virion and the genome emptied virion connectors. The negative and positive electrostatic potentials are colored red and blue, respectively. The exposed hydrophobic surface of the mature and the genome-emptied virion connectors are outlined with black lines. The scale bar is 5 nm.

(c) Left to Right: ribbon diagrams showing the salt bridge pairs between the capsid shell and the connector of the mature virion and its comparisons with those in the prohead and the genome-emptied virion. The relative positions of connectors and capsid shells are the same as in “Figure 6a”. The side chains of Arg94 and Glu103 in the connector and Lys297 and Asp298 in the capsid shell are shown in sticks. Arg94 and Glu103 form salt bridges with Asp298 and Lys297 only in the mature virion.

## Supplementary Figure 5

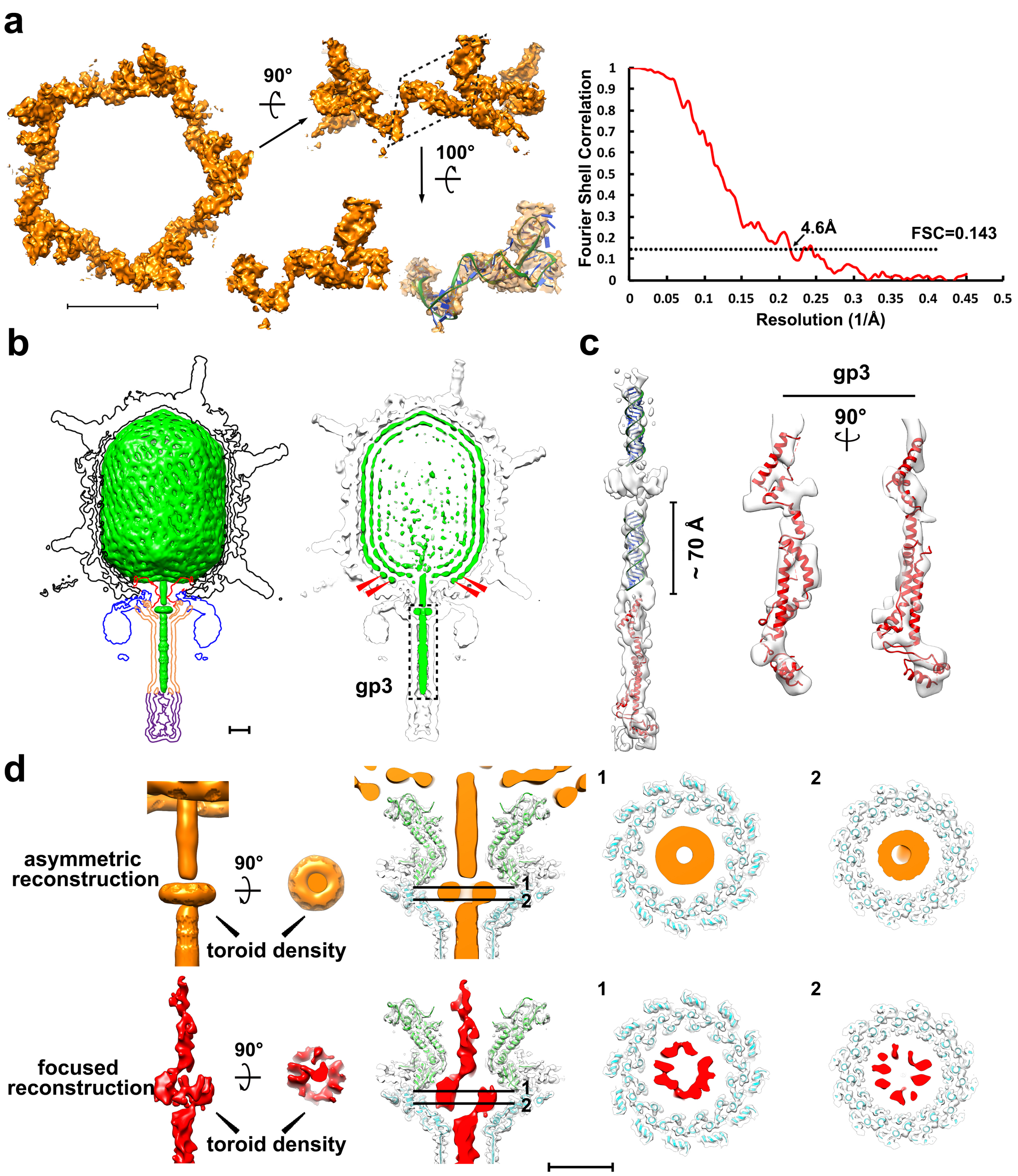

**Supplementary Figure 5. The pRNA structure and the genome organization.**
(a) Cryo-EM density map of the pRNA ring from the focused refinements. Left: top view. Middle top: side view. One protomer of the pRNA ring is outlined with a frame box. Middle bottom: Zoom-ins showing densities of one pRNA protomer. The RNA duplex features could be clearly identified in map. Right: Gold standard Fourier shell correlation (FSC) curve of the pRNA ring reconstruction. The scale bar represents 5 nm.
(b) Left: Surface-shadowed diagrams showing the genome organization in the C1 reconstruction of the mature virion. The outlines of the capsid and the tail components are shown in different colors (capsid: black, connector: red, tail lower collar: orange, tail spikes: blue, tail knob: purple, genome: green); right: A thin section of the genome showing the genome layers. Positions of the two concentric DNA rings around the wide end of the connector are indicated by arrows.
(c) A zoom-in showing the density in the tail tube. The ribbon models of the terminal protein gp3 (red) and a piece of DNA fragment (blue) are fitted in the density. The terminal protein gp3 model was obtained from the MDFF fitting.
(d) Toroid density in the collar cavity from the C1 reconstruction and the focused refinement. Left: Side view and the top view of the toroid density from the C1 reconstruction (orange) and the focused refinement (red); middle and right: Thin sections of the toroid density showing no direct contact between the density and the collar. The connector (green) and lower collar (cyan) models are shown in the density map.

## Supplementary Figure 6

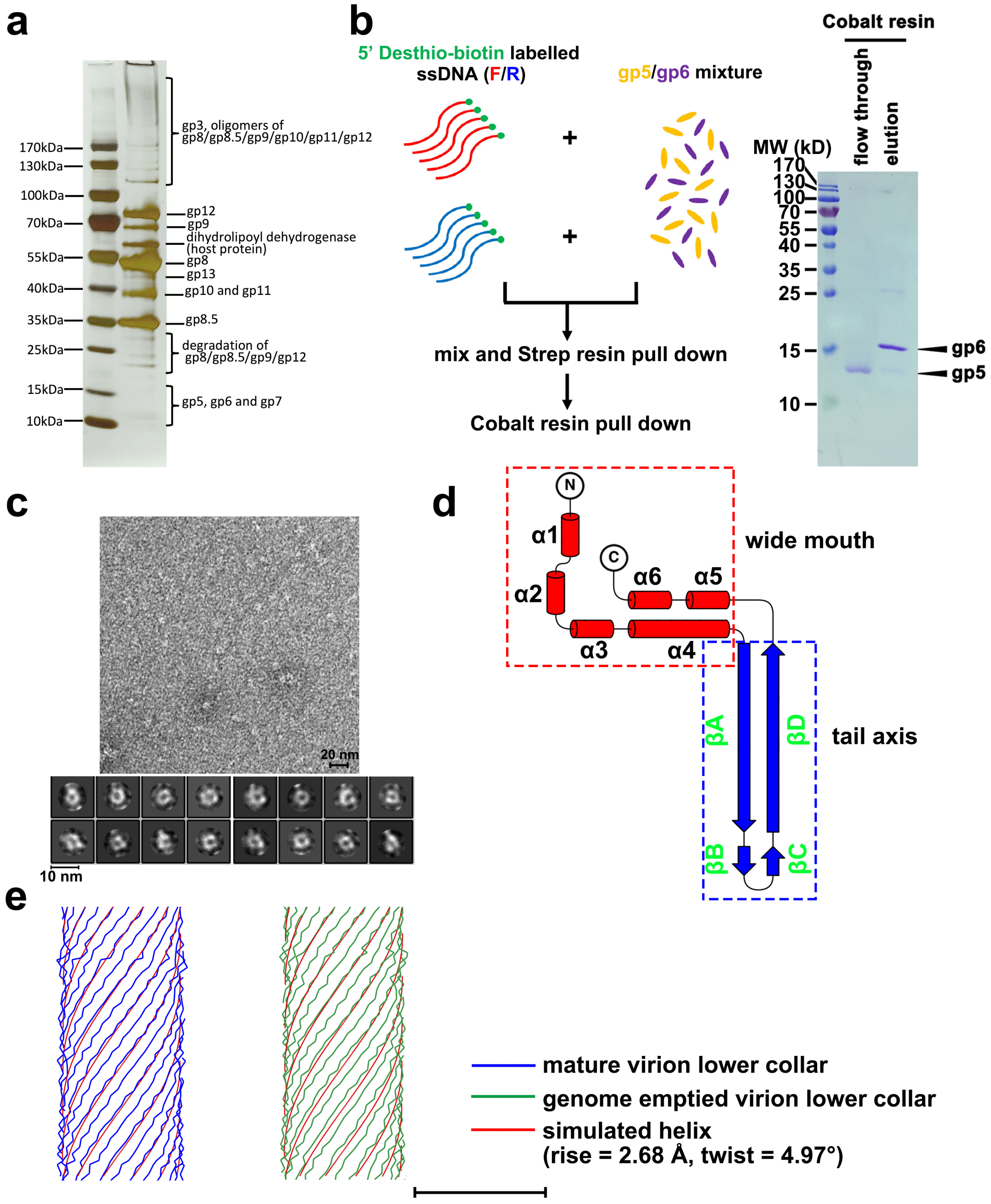

**Supplementary Figure 6. SDS-PAGE gel and mass spectrometric analysis of the mature phage particles, analysis of the gp5 and gp6 pull downs with ssDNA fragments, and structure of the lower collar.**

(a) A silver stained SDS-PAGE gel of the mature phage particles showing the structure components of the mature virion. The purified mature phage sample was boiled for 5 minutes in presence of 2% SDS. The major bands were cut and sent for mass spectrometric analysis. Mass spectrometric results from the bands on the gel are listed at the right side of the bands (see also Supplementary Table 5).

(b) Left: a schematic diagram showing the preparation of the gp5-gp6-ssDNA complex; right: SDS-PAGE gel analysis of gp5 and gp6 pulled down by two desthiobiotin-labeled 59 nt ssDNA fragments, of which the sequences are the same as the forward and reverse strands of the right end 21-79 bp of the ϕ29 genome, respectively. The desthiobiotin-label ssDNAs was added to the purified gp5 and gp6 proteins to pull down the interaction proteins using Strep resins. The bands of gp5 and gp6 were marked.

(c) Top: Negative staining micrograph of the gp5-gp6-ssDNA complex. The scale bar is 20 nm; bottom: 2D classification analysis of the gp5-gp6-ssDNA complex showing a ring-shaped structure. The scale bar is 10 nm.

(d) A diagram showing the topology of gp11.

(e) Comparisons of the lower collar tube structures with a simulated helix, showing that the lower collar tube of the mature virion is closely approximate to the simulated helix, whereas the bottom end of the lower collar tube from the genome emptied virion is off from the simulated helix, indicating conformational changes in the distal end of the tail tube upon genome release.

## Supplementary Figure 7

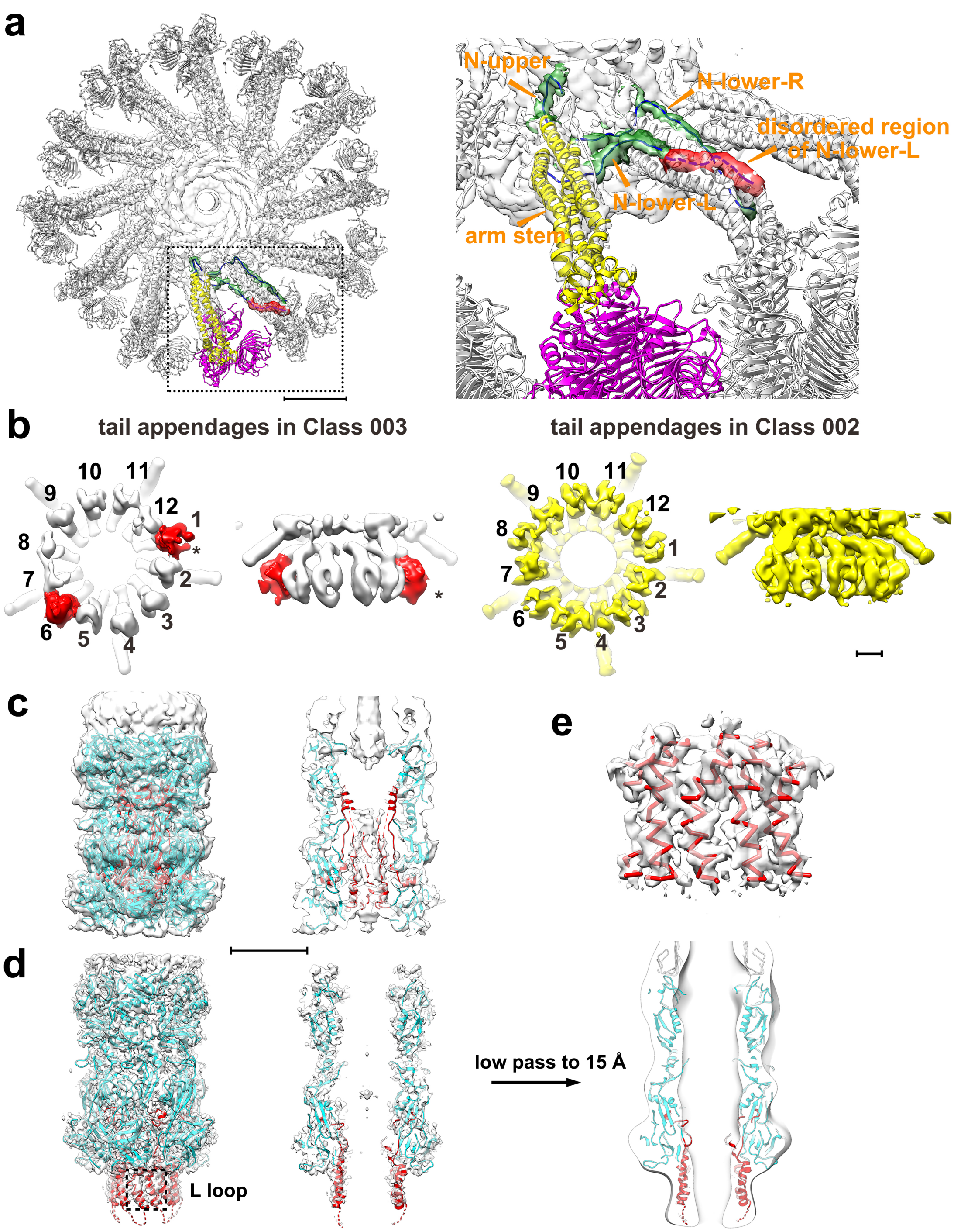

**Supplementary Figure 7. Structures of the tail appendages and tail knob.**

(a) Low pass filtered map (to 5 Å) showing the N termini of the tail spikes. The N termini of one tail appendage are colored green. The disordered region of the N-lower-L is highlighted red and is represented with dashed blue lines in the ribbon model. Two lower N termini of each tail appendage at the lower position (N-lower-L, N-lower-R) extend to and glue with the three helix coiled-coil (yellow) of a neighboring tail spike to form the arm. The scale bar is 5 nm.

(b) Surface shadowed diagrams showing the "up" and "down" conformations of two distinct appendage groups. The appendages adopting an "up" conformation are colored red.

(c, d) The tail knob models (gp9) shown in the mature (c) and the genome emptied (d) virion EM maps. The hydrophobic L-loops are colored red and the rest of the tail knob is colored cyan. The disordered region of the L-loops (residue 440-455) is represented by dashed red lines. (d) right: the disordered region of the L-loop becomes visible in the low-pass filtered map (to 15 Å).

(e) A zoom-in showing the L-loops forming an alpha helix barrel upon genome release. The scale bar represents 5 nm.

## Supplementary Figure 8

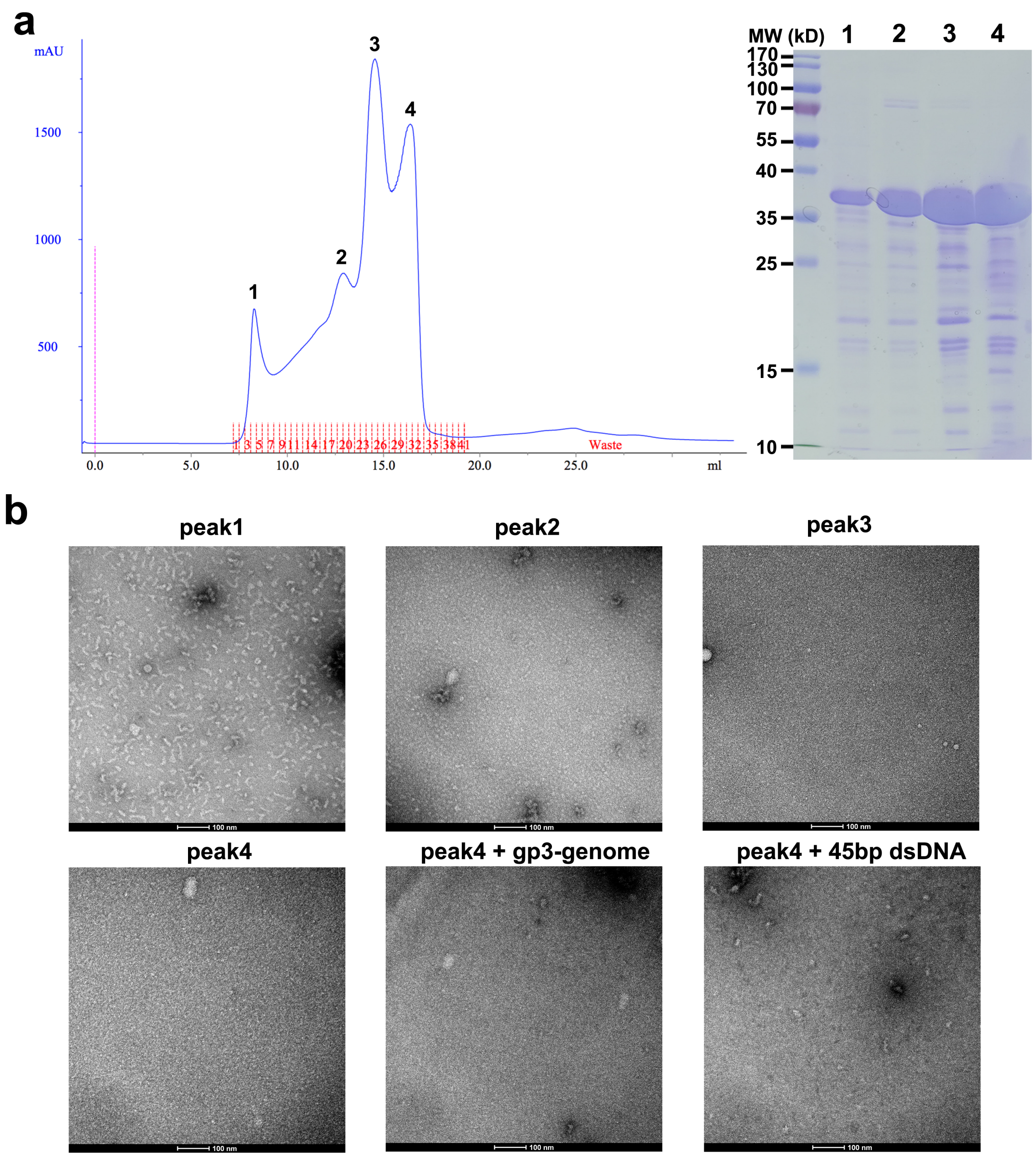

**Supplementary Figure 8. Analysis of the in vitro assembly of gp11.**

(a) Size exclusion chromatography elution profile of gp11 and SDS-PAGE gel analyses of the elution fractions.

(b) Negative staining analyses of gp11 fractions and gp11 mixed with a 45 bp dsDNA fragment or the purified gp3-genome. The scale bar represents 100 nm.

## Supplementary Figure 9

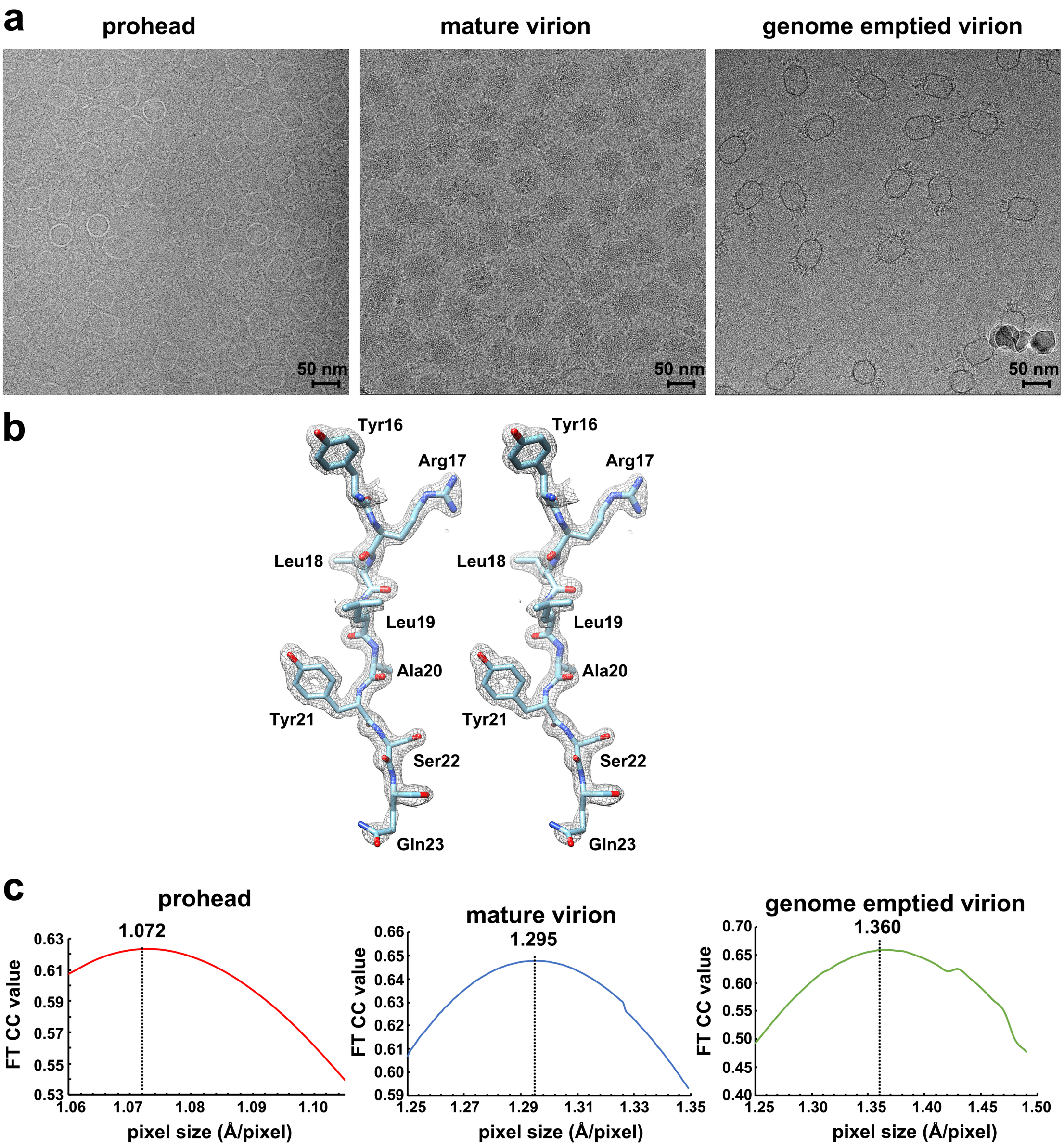

**Supplementary Figure 9. Representative raw micrographs of different particles, representative model and density map of gp8.5-N and magnification calibration of different EM maps.**

(a) Representative micrographs for the prohead, the mature virion and the genome emptied virion.

(b) A stereo diagram showing a representative portion of the gp8.5 N crystal structure. The polypeptide chain is shown in sticks. The *2Fo-Fc* electron density map is contoured at 0.057 e/$Å^3$.

(c) Magnification calibration of different EM maps using crystal structures. Please refer to Methods for details.

## Supplementary Table 1. Cryo-EM data collection and model statistics

| | Prohead | | Mature virion | | | Genome emptied virion | | |
|---|---|---|---|---|---|---|---|---|
| **Data collection** | | | | | | | | |
| EM equipment | FEI Titan Krios | | | | | | | |
| Voltage (kV) | 300 | | | | | | | |
| Detector | Kodak SO-163 films | | K2 camera | | | Falcon II camera | | |
| Pixel size (Å) | 1.072 | | 1.295 | | | 1.360 | | |
| Electron dose (e/Å$^2$) | 25 | | 40 | | | 50 | | |
| Defocus range ($\mu$m) | 1.4-2.8 | | 1.0-3.3 | | | 1.0-2.5 | | |
| | Capsid[a] | connector | Capsid[a] | connector | collar complex[b] | Capsid[a] | connector | collar complex[b] |
| **Reconstruction** | | | | | | | | |
| Software | EMAN, Relion & jspr.py | | | | | | | |
| Number of used particles | 18230 | 18230 | 36730 | 36730 | 36730 | 44059 | 37761 | 31478 |
| Final Resolution (Å) | 3.6 | 3.8 | 3.2 | 3.4 | 3.3 | 3.2 | 3.6 | 4.0 |
| **Model building** | | | | | | | | |
| Software | Coot | | | | | | | |
| **Refinement** | | | | | | | | |
| Software | *phenix.real_space_refine* & RosettaCM | | | | | | | |
| Symmetry | 5 | 12 | 5 | 12 | 12 | 5 | 12 | 12 |
| Map CC | 0.692 | 0.610 | 0.709 | 0.577 | 0.736 | 0.685 | 0.614 | 0.750 |
| **Validation** | | | | | | | | |
| R.M.S deviations | | | | | | | | |
| Bonds length(Å) | 0.016 | 0.006 | 0.015 | 0.004 | 0.005 | 0.014 | 0.007 | 0.010 |
| Bonds Angle (˚) | 1.42 | 1.27 | 1.54 | 0.86 | 0.939 | 1.53 | 0.879 | 1.06 |
| Ramachandran plot statistics (%) | | | | | | | | |
| Preferred | 89.7 | 84.5 | 90.5 | 93.5 | 93.1 | 90.3 | 93.5 | 90.2 |
| Allowed | 8.0 | 14.7 | 7.5 | 6.5 | 6.8 | 7.6 | 6.5 | 9.8 |
| Outlier | 2.3 | 0.8 | 2.0 | 0.0 | 0.1 | 2.1 | 0.0 | 0.0 |
| Molprobity score | 1.6 | 2.2 | 1.6 | 1.7 | 1.8 | 2.0 | 1.9 | 2.1 |

[a]The major capsid proteins and the N termini of the head fiber proteins are used for the statistics.
[b]The connector, lower collar and the N terminal and arm stem domains of the tail spikes are used for the statistics.

## Supplementary Table 2. Comparisons of the capsomer structures in the mature virion head

**Structural comparisons among different hexameric capsomeres**

| capsomer pair | minimum r.m.s.d. | capsomer pair | minimum r.m.s.d.[a] |
|---|---|---|---|
| **H6-H1** | 1.0 | **H3-H2** | 3.4 |
| **H5-H1** | 0.6 | **H6-H3** | 3.6 |
| **H4-H1** | 3.6 | **H5-H3** | 3.4 |
| **H3-H1** | 3.6 | **H4-H3** | 0.4 |
| **H2-H1** | 0.6 | **H6-H4** | 3.6 |
| **H6-H2** | 1.1 | **H5-H4** | 3.4 |
| **H5-H2** | 0.4 | **H6-H5** | 1.2 |
| **H4-H2** | 3.4 | | |
| Average r.m.s.d. among hexameric capsomeres: 2.2 Å | | | |

**Structural comparison among different pentameric capsomeres**

| capsomer pair | minimum r.m.s.d.[a] |
|---|---|
| **P3-P1** | 0.7 |
| **P2-P1** | 0.7 |
| **P3-P2** | 0.4 |
| Average r.m.s.d. among pentameric capsomeres: 0.6 Å | |

**Structural comparisons of each hexmeric capsomer with its rotation equivalents**

| capsomer | 0° | 60°[a] | 120°[a] | 180°[a] | 240°[a] | 300°[a] |
|---|---|---|---|---|---|---|
| **H6** | - | 2.2 | 1.4 | 2.0 | 1.4 | 2.2 |
| **H5** | - | 1.9 | 0.9 | 1.8 | 0.9 | 1.9 |
| **H4** | - | 5.8 | 5.8 | 0.7 | 5.8 | 5.8 |
| **H3** | - | 5.8 | 5.8 | 0.7 | 5.8 | 5.8 |
| **H2** | - | 1.9 | 0.9 | 1.8 | 0.9 | 1.9 |
| **H1** | - | 2.1 | 0.3 | 2.1 | 0.3 | 2.1 |

**Structural comparisons of each pentameric capsomer with its rotation equivalents**

| capsomer | 0° | 72°[a] | 144°[a] | 216°[a] | 288°[a] |
|---|---|---|---|---|---|
| **P3** | - | 0.5 | 0.4 | 0.4 | 0.5 |
| **P2** | - | 0.5 | 0.4 | 0.4 | 0.5 |

[a]r.m.s.ds. in Angstrom. The Cα atoms of the HK97 domains (residues 62-347) are used for the alignments and r.m.s.d. calculations.

# Supplementary Table 3. Summary of the salt bridges in the mature virion structure
## Salt bridges in the mature virion head

| Residue-atom | Chain ID* | Residue-atom | Chain ID* | Distance (Å) | Residue-atom | Chain ID* | Residue-atom | Chain ID* | Distance (Å) |
|---|---|---|---|---|---|---|---|---|---|
| **Intra-salt bridges (pentameric capsomer: P3)[a]** | | | | | | | | | |
| Lys223-NZ | P3-A | Asp254-OD1 | P3-B | 3.1 | Lys215-NZ | P3-A | Asp262-OD1 | P3-B | 3.6 |
| Lys223-NZ | P3-A | Asp254-OD2 | P3-B | 3.0 | Lys215-NZ | P3-A | Asp262-OD2 | P3-B | 2.8 |
| Arg218-NH1 | P3-A | Glu256-OE1 | P3-B | 2.8 | Asp298-OD2 | P3-A | Arg75-NH1 | P3-B | 3.3 |
| Arg218-NH2 | P3-A | Glu256-OE2 | P3-B | 3.0 | Asp298-OD2 | P3-A | Arg75-NH2 | P3-B | 2.9 |
| Arg218-NH1 | P3-A | Asp260-OD2 | P3-B | 3.3 | Glu107-OE2 | P3-A | Arg136-NH2 | P3-B | 2.8 |
| Arg218-NH2 | P3-A | Asp260-OD2 | P3-B | 2.9 | Arg222-NH2 | P3-A | Glu256-OE2 | P3-B | 3.1 |
| Lys215-NZ | P3-A | Glu258-OE1 | P3-B | 2.8 | Lys192-NZ | P3-A | Glu16-OE2 | P3-B | 2.8 |
| Lys215-NZ | P3-A | Glu258-OE2 | P3-B | 3.8 | | | | | |
| **Intra-salt bridges (hexameric capsomer: H5)[b]** | | | | | | | | | |
| Arg95-NH1 | H5-A | Asp67-OD2 | H5-B | 2.9 | Glu99-OE2 | H5-A | Arg75-NH2 | H5-B | 2.8 |
| Lys215-NZ | H5-A | Asp262-OD1 | H5-B | 3.6 | Lys192-NZ | H5-A | Glu16-OE1 | H5-B | 2.9 |
| Lys215-NZ | H5-A | Asp262-OD2 | H5-B | 2.7 | Glu107-OE2 | H5-A | Arg136-NH2 | H5-B | 2.9 |
| Lys215-NZ | H5-A | Glu258-OE1 | H5-B | 2.8 | Lys223-NZ | H5-A | Asp254-OD1 | H5-B | 3.2 |
| Lys215-NZ | H5-A | Glu258-OE2 | H5-B | 3.8 | Lys223-NZ | H5-A | Asp254-OD2 | H5-B | 3.0 |
| Arg222-NH1 | H5-A | Glu256-OE1 | H5-B | 2.7 | Arg272-NH1 | H5-A | Asp260-OD1 | H5-B | 3.0 |
| Arg222-NH2 | H5-A | Glu256-OE2 | H5-B | 3.0 | Asp298-OD2 | H5-A | Arg75-NH1 | H5-B | 3.0 |
| Arg218-NH2 | H5-A | Asp260-OD2 | H5-B | 2.9 | | | | | |
| **Inter-capsomer salt bridges (P3-H4-H5, pentamer-hexamer-hexamer)[c]** | | | | | | | | | |
| Arg122-NH2 | P3-C | Glu113-OE2 | H5-E | 2.7 | Arg316-NH1 | P3-C | Glu123-OE2 | H4-E | 3.6 |
| Glu121-OE1 | P3-B | Arg316-NH1 | H5-A | 2.8 | Arg316-NH2 | P3-C | Glu123-OE2 | H4-E | 3.9 |
| Glu121-OE1 | P3-B | Arg316-NH2 | H5-A | 3.0 | Glu113-OE1 | P3-A | Lys362-NZ | H4-F | 2.7 |
| Arg313-NH1 | P3-D | Glu310-OE1 | H5-F | 3.6 | Lys362-NZ | P3-B | Glu113-OE1 | H4-E | 2.8 |
| Arg313-NH1 | P3-D | Glu310-OE2 | H5-F | 2.9 | Lys362-NZ | P3-B | Asp111-OD2 | H4-E | 4.0 |
| Glu116-OE2 | P3-B | Arg122-NH1 | H5-F | 3.1 | Arg122-NH2 | P3-B | Glu116-OE2 | H4-E | 3.9 |
| Lys308-NZ | P3-C | Asp147-OD1 | H5-A | 3.0 | Arg313-NH2 | P3-C | Glu310-OE1 | H4-F | 3.5 |
| Lys308-NZ | P3-C | Asp147-OD2 | H5-A | 2.9 | Arg313-NH2 | P3-C | Glu310-OE2 | H4-F | 2.9 |
| Glu113-OE1 | P3-B | Lys362-NZ | H5-F | 3.4 | Lys308-NZ | P3-B | Asp147-OD1 | H4-F | 3.2 |
| Glu113-OE2 | P3-B | Lys362-NZ | H5-F | 3.4 | Lys308-NZ | P3-B | Asp147-OD2 | H4-F | 2.8 |
| Glu310-OE1 | P3-C | Arg313-NH2 | H5-A | 3.9 | Glu121-OE1 | P3-A | Arg316-NH1 | H4-A | 2.9 |
| Glu310-OE2 | P3-C | Arg313-NH2 | H5-A | 3.0 | Glu121-OE2 | P3-A | Arg316-NH2 | H4-A | 3.9 |
| Arg316-NH1 | P3-D | Glu123-OE2 | H5-E | 3.3 | Glu114-OE1 | P3-A | Arg316-NH2 | H4-A | 2.9 |
| Arg316-NH2 | P3-D | Glu123-OE2 | H5-E | 3.8 | Glu310-OE1 | P3-B | Arg313-NH2 | H4-A | 3.8 |
| Arg316-NH1 | P3-C | Glu121-OE1 | H4-E | 3.4 | Glu310-OE2 | P3-B | Arg313-NH2 | H4-A | 2.9 |
| Arg316-NH2 | P3-C | Glu121-OE1 | H4-E | 3.2 | Lys308-NZ | H5-A | Asp147-OD1 | H4-F | 3.8 |
| Arg122-NH1 | H5-A | Glu113-OE1 | H4-D | 3.0 | Lys308-NZ | H5-A | Asp147-OD2 | H4-F | 2.9 |
| Arg122-NH2 | H5-A | Glu113-OE1 | H4-D | 2.9 | Glu310-OE1 | H5-A | Arg313-NH2 | H4-F | 3.5 |
| Arg122-NH1 | H5-A | Glu116-OE2 | H4-D | 2.9 | Glu310-OE2 | H5-A | Arg313-NH2 | H4-F | 2.9 |
| Glu98-OE1 | H5-A | Arg68-NH2 | H4-F | 3.0 | Arg313-NH2 | H5-B | Glu310-OE2 | H4-E | 2.9 |
| Glu98-OE2 | H5-A | Arg68-NH2 | H4-F | 3.8 | Asp147-OE1 | H5-B | Lys308-NZZ | H4-E | 3.4 |
| Lys362-NZ | H5-A | Asp111-OD1 | H4-D | 3.9 | Asp147-OE2 | H5-B | Lys308-NZ | H4-E | 2.8 |
| Lys362-NZ | H5-A | Asp111-OD2 | H4-D | 2.8 | Glu121-OE1 | H5-F | His141-ND1 | H4-A | 3.5 |
| Glu113-OE1 | H5-F | Arg438-NH1 | H4-E | 3.1 | Glu113-OE1 | H5-F | Arg122-NH2 | H4-E | 3.5 |
| Glu113-OE1 | H5-F | Arg438-NH2 | H4-E | 3.0 | | | | | |
| **Inter-capsomer salt bridges (H2-H3-H4, hexamer-hexamer-hexamer)[d]** | | | | | | | | | |
| Glu310-OE1 | H4-D | Arg313-NH2 | H3-E | 2.9 | Arg316-NH2 | H4-D | Glu121-OE2 | H2-C | 3.2 |
| Glu310-OE2 | H4-D | Arg313-NH2 | H3-E | 3.5 | Asp60-OD1 | H4-D | Arg438-NH1 | H2-D | 2.8 |
| Glu113-OE2 | H4-C | Arg122-NH1 | H3-D | 2.8 | Glu113-OE1 | H4-B | Arg122-NH1 | H2-D | 2.8 |
| Glu113-OE2 | H4-C | Arg122-NH2 | H3-D | 2.9 | Glu310-OE1 | H4-C | Arg313-NH2 | H2-E | 3.6 |
| Lys308-NZ | H4-D | Asp147-OD1 | H3-E | 3.4 | Glu310-OE2 | H4-C | Arg313-NH2 | H2-E | 2.8 |
| Lys308-NZ | H4-D | Asp147-OD2 | H3-E | 2.8 | Lys362-NZ | H4-C | Asp111-OD1 | H2-C | 3.2 |
| Arg316-NH1 | H4-E | Glu121-OE1 | H3-C | 2.8 | Lys362-NZ | H4-C | Asp111-OD2 | H2-C | 2.8 |
| Arg316-NH2 | H4-E | Glu121-OE1 | H3-C | 2.9 | Lys308-NZ | H4-C | Asp147-OD1 | H2-E | 4.0 |
| Lys362-NZ | H4-D | Glu113-OE1 | H3-C | 2.8 | Lys308-NZ | H4-C | Asp147-OD1 | H2-E | 2.9 |
| Asp147-OD1 | H4-E | Lys308-NZ | H3-D | 3.1 | Arg122-NH2 | H4-C | Glu113-OE1 | H2-C | 2.9 |
| Asp147-OD2 | H4-E | Lys308-NZ | H3-D | 2.9 | Asp147-OD1 | H4-D | Lys308-NZ | H2-D | 3.3 |
| Arg313-NH2 | H4-E | Glu310-OE1 | H3-D | 3.7 | Asp147-OD2 | H4-D | Lys308-NZ | H2-D | 2.9 |
| Arg313-NH2 | H4-E | Glu310-OE2 | H3-D | 2.9 | Arg313-NH2 | H4-D | Glu310-OE1 | H2-D | 3.6 |
| Glu116-OE2 | H4-C | Arg122-NH1 | H3-D | 3.0 | Arg313-NH2 | H4-D | Glu310-OE2 | H2-D | 2.9 |
| Arg438-NH1 | H4-D | Glu113-OE2 | H3-C | 3.1 | Glu121-OE1 | H4-B | His141-ND1 | H2-C | 3.7 |
| Glu98-OE1 | H4-D | Arg68-NH1 | H3-E | 3.3 | Glu121-OE1 | H4-B | Arg316-NH1 | H2-E | 3.7 |
| Arg316-NH1 | H4-D | Glu121-OE1 | H2-C | 3.5 | Glu121-OE1 | H4-B | Arg316-NH2 | H2-E | 2.9 |
| Glu114-OE2 | H3-D | Lys362-NZ | H2-C | 3.0 | Glu310-OE2 | H3-E | Arg313-NH2 | H2-D | 2.8 |
| Glu113-OE1 | H3-D | Arg122-NH2 | H2-C | 2.7 | Arg68-NH1 | H3-F | Glu98-OE1 | H2-C | 3.3 |
| Glu121-OE1 | H3-D | His141-ND1 | H2-E | 3.7 | Arg68-NH2 | H3-F | Glu98-OE2 | H2-C | 3.8 |
| Lys362-NZ | H3-E | Asp111-OD2 | H2-B | 3.6 | Arg313-NH2 | H3-F | Glu310-OE1 | H2-C | 3.7 |
| Lys362-NZ | H3-E | Glu113-OE2 | H2-B | 2.8 | Arg313-NH2 | H3-F | Glu310-OE2 | H2-C | 2.8 |
| Lys308-NZ | H3-E | Asp147-OD1 | H2-D | 3.5 | Arg316-NH1 | H3-F | Glu121-OE1 | H2-B | 2.8 |
| Lys308-NZ | H3-E | Asp147-OD2 | H2-D | 2.8 | Arg316-NH2 | H3-F | Glu131-OE1 | H2-B | 3.0 |
| Arg122-NH2 | H3-E | Glu116-OE2 | H2-B | 3.7 | Asp147-OD1 | H3-F | Lys308-NZ | H2-C | 3.3 |
| Glu310-OE1 | H3-E | Arg313-NH2 | H2-D | 3.7 | Asp147-OD2 | H3-F | Lys308-NZ | H2-C | 2.9 |

*The five gp8 molecules of each pentameric capsomer are labeled from A to E. The six gp8 molecules of each hexameric capsomer are labeled from A to F. The twelve gp10 molecules of the connector are labeled from A to L. The twelve gp11 molecules of the lower collar

are labeled from A to L. The three gp12* molecules of each tail spike are labeled from A to C.
[a]Similar salt bridges were observed for P1 and P2.
[b]Similar salt bridges were observed for H1, H2, H3, H4 and H6.
[c]Similar salt bridges were observed for P2-H2-H3, P3-H5-H6 and P2-H1-H2.
[d]Similar salt bridges were observed for H3-H4-H5.

# Salt bridges in the mature virion tail

| | | | | | | | | | |
|---|---|---|---|---|---|---|---|---|---|
| **Salt bridges between the connector (Co-A) and the lower collar (Lo-A)[e]** | | | | | | | | | |
| Asp165-OD2 | Co-A | Arg62-NH1 | Lo-A | 4.0 | | | | | |
| **Salt bridges within the lower collar (between Lo-A and Lo-B)[f]** | | | | | | | | | |
| Arg122-NH2 | Lo-A | Asp238-OD2 | Lo-B | 3.6 | Glu284-OE2 | Lo-A | Arg276-NH2 | Lo-B | 3.4 |
| Asp161-OD2 | Lo-A | Arg202-NH2 | Lo-B | 3.6 | Lys89-NZ | Lo-A | Glu94-OE2 | Lo-B | 2.6 |
| Glu94-OE2 | Lo-A | Arg271-NZ | Lo-B | 3.7 | Glu77-OE2 | Lo-A | Arg57-NH1 | Lo-B | 3.8 |
| Glu284-OE1 | Lo-A | Arg276-NH2 | Lo-B | 2.8 | Glu77-OE2 | Lo-A | Arg57-NH2 | Lo-B | 3.5 |
| **Salt bridges between the lower collar (Lo-A) and the tail spike arms (SA1, SA12)[g]** | | | | | | | | | |
| Glu69-OE1 | Lo-A | Lys38-NZ | SA1-B | 3.7 | Arg9-NH1 | Lo-A | Glu37-OE2 | SA12-B | 3.1 |
| Glu69-OE2 | Lo-A | Lys38-NZ | SA1-B | 2.8 | Arg9-NH2 | Lo-A | Glu37-OE1 | SA12-B | 3.7 |
| **Salt bridges in one tail spike arm (SA1)[h]** | | | | | | | | | |
| Glu31-OE1 | SA1-C | Arg23-NH1 | SA1-B | 3.3 | Lys41-NZ | SA1-C | Glu22-OE2 | SA1-B | 3.0 |
| **Salt bridges between the tail spike arms (SA1 with SA2, SA12)[i]** | | | | | | | | | |
| Glu55-OE1 | SA1-C | Arg9-NH1 | SA2-C | 2.8 | Glu37-OE2 | SA1-B | Arg23-NH2 | SA2-B | 2.6 |
| Glu55-OE1 | SA1-C | Arg9-NH2 | SA2-C | 3.6 | Glu37-OE2 | SA1-B | Arg23-NH1 | SA2-B | 3.8 |
| Arg23-NH1 | SA1-C | Glu22-OE1 | SA12-A | 3.2 | Glu22-OE1 | SA1-A | Arg23-NH1 | SA2-C | 3.2 |
| Arg23-NH1 | SA1-C | Glu22-OE2 | SA12-A | 2.9 | Glu22-OE2 | SA1-A | Arg23-NH1 | SA2-C | 2.9 |

[e]Similar salt bridges were observed between other subunit pairs of the connector and the lower collar.
[f]Similar salt bridges were observed between other subunit pairs of the lower collar.
[g]Similar salt bridges were observed between other subunit pairs of the lower collar and the tail spike arms.
[h]Similar salt bridges were observed in other tail spike arms.
[i]Similar salt bridges were observed between other tail spike arms.

# Supplementary Table 4. Structural comparisons between the prohead, mature virion head and genome-emptied virion head

| capsomer | prohead-mature[a] | | mature-genome emptied[a] | | prohead-genome emptied[a] | |
|---|---|---|---|---|---|---|
| | capsid superposition | Individual capsomer superposition | capsid superposition | Individual capsomer superposition | capsid superposition | Individual capsomer superposition |
| **H6** | 1.2 | 0.6 | 1.1 | 0.5 | 0.6 | 0.6 |
| **H5** | 0.4 | 0.4 | 0.8 | 0.6 | 0.9 | 0.6 |
| **H4** | 0.5 | 0.4 | 1.2 | 0.7 | 1.0 | 0.6 |
| **H3** | 0.6 | 0.4 | 1.2 | 0.7 | 1.0 | 0.6 |
| **H2** | 0.6 | 0.4 | 0.8 | 0.6 | 0.9 | 0.6 |
| **H1** | 0.5 | 0.4 | 0.6 | 0.6 | 0.6 | 0.5 |
| **P1** | 0.8 | 0.5 | 1.0 | 0.4 | 0.6 | 0.5 |
| **P2** | 0.9 | 0.4 | 1.1 | 0.4 | 0.6 | 0.5 |
| **P3** | 0.9 | 0.4 | 1.1 | 0.5 | 0.5 | 0.5 |

| | equator area[a] | end icosahedral cap[a] | overall[a] |
|---|---|---|---|
| **prohead-mature** | 0.5 | 0.8 | 0.7 |
| **emptied-mature** | 1.2 | 0.9 | 1.0 |
| **prohead-emptied** | 1.0 | 0.7 | 0.8 |

| | Prohead | Mature virion | Genome emptied virion |
|---|---|---|---|
| **Center of gravity of the capsid equator area (z only, $h_1$, Å)** | -7.7 | 2.8 | 3.5 |
| **Center of gravity of the connector neck (z only, $h_2$, Å)** | -235.4 | -237.7 | -235.2 |
| **Center of gravity of the capsomer H6 (z only, $h_3$, Å)** | -210.1 | -200.5 | -198.9 |
| **Relative distance connector-capsid ($h_1 - h_2$, Å)** | 227.7 | 240.5 | 238.7 |
| **Relative distance H6-capsid ($h_1 - h_3$, Å)** | 202.4 | 203.3 | 202.4 |
| **Relative distance to the prohead connector (Å)** | - | 12.8 | 11.0 |

[a]r.m.s.d. in Angstrom. The Cαs of the HK97 domains (residues 62-347) are used for the alignments and r.m.s.d. calculations.

**Supplementary Table 5. Mass spectrometric analysis of the purified mature $\phi$29 virion**

| Protein accession | Molecular weight (kDa) | Total unique peptide count | Annotation |
|---|---|---|---|
| P68930 | 33.8 | 25 | Proximal tail tube connector protein (gp11) |
| B3VMP4 | 29.5 | 21 | Capsid fiber protein (gp8.5) |
| P04331 | 67.5 | 19 | Tail knob protein (gp9) |
| P20345 | 92.0 | 18 | Appendage protein (gp12) |
| P13849 | 49.8 | 17 | Major capsid protein (gp8) |
| P03681 | 31.0 | 8 | Primer terminal protein (gp3) |
| P04332 | 35.9 | 6 | Portal protein (gp10) |
| P15132 | 40.9 | 6 | Morphogenesis protein 1 (gp13) |
| Q38504 | 13.3 | 5 | Single-stranded DNA-binding protein (gp5) |
| P11187 | 28.0 | 5 | Endolysin (gp15) |
| P13848 | 11.3 | 4 | Capsid assembly scaffolding protein (gp7) |
| P03685 | 12.0 | 3 | Histone-like protein (gp6) |
| P11188 | 15.0 | 1 | Antiholin (gp14) |
